# Potential Novel Serum Metabolic Markers Associated With Progression of Prediabetes to Overt Diabetes in a Chinese Population

**DOI:** 10.3389/fendo.2021.745214

**Published:** 2022-01-05

**Authors:** Meng Ren, Diao zhu Lin, Zhi Peng Liu, Kan Sun, Chuan Wang, Guo juan Lao, Yan qun Fan, Xiao yi Wang, Jing Liu, Jie Du, Guo bin Zhu, Jia huan Wang, Li Yan

**Affiliations:** ^1^ Department of Endocrinology, Sun Yat-Sen Memorial Hospital, Sun Yat-Sen University, Guangzhou, China; ^2^ Biotree-Shanghai, Focus Dream Park, Shanghai, China

**Keywords:** prediabetes, diabetes, metabolites, prediction, clinical

## Abstract

**Background:**

Identifying the metabolite profile of individuals with prediabetes who turned to type 2 diabetes (T2D) may give novel insights into early T2D interception. The purpose of this study was to identify metabolic markers that predict the development of T2D from prediabetes in a Chinese population.

**Methods:**

We used an untargeted metabolomics approach to investigate the associations between serum metabolites and risk of prediabetes who turned to overt T2D (n=153, mean follow up 5 years) in a Chinese population (REACTION study). Results were compared with matched controls who had prediabetes at baseline [age: 56 ± 7 years old, body mass index (BMI): 24.2 ± 2.8 kg/m2] and at a 5-year follow-up [age: 61 ± 7 years old, BMI: 24.5 ± 3.1 kg/m2]. Confounding factors were adjusted and the associations between metabolites and diabetes risk were evaluated with multivariate logistic regression analysis. A 10-fold cross-validation random forest classification (RFC) model was used to select the optimal metabolites panels for predicting the development of diabetes, and to internally validate the discriminatory capability of the selected metabolites beyond conventional clinical risk factors.

**Findings:**

Metabolic alterations, including those associated with amino acid and lipid metabolism, were associated with an increased risk of prediabetes progressing to diabetes. The most important metabolites were inosine [odds ratio (OR) = 19.00; 95% confidence interval (CI): 4.23-85.37] and carvacrol (OR = 17.63; 95% CI: 4.98-62.34). Thirteen metabolites were found to improve T2D risk prediction beyond eight conventional T2D risk factors [area under the curve (AUC) was 0.98 for risk factors + metabolites vs 0.72 for risk factors, P < 0.05].

**Interpretations:**

Use of the metabolites identified in this study may help determine patients with prediabetes who are at highest risk of progressing to diabetes.

## Introduction

Prediabetes is an intermediate metabolic state of hyperglycemia in which the serum glucose level is higher than normal, but lower than the diagnostic threshold for diabetes. Prediabetes can be considered a heterogeneous, subclinical form of diabetes. The prevalence of prediabetes has increased significantly in recent decades, and the estimated standardized prevalence of prediabetes has reached 35.7% in the Chinese adult population (1). Individuals with prediabetes have a higher risk of developing diabetes, and the lifetime conversion rate to T2D is as high as 74% (2). Given the availability of lifestyle interventions that are effective at preventing or delaying the onset of T2D, early identification of persons with prediabetes is important. A number of traditional markers are used to estimate the risk of T2D in normal individuals, such as fasting plasma glucose and glycated hemoglobin A1c (HbA1c) (3). However, most of these markers fail to capture the complexity of the etiology of prediabetes, and thus are limited with respect to detecting early metabolic abnormalities that may occur years or even decades before the onset/diagnosis of overt diabetes.

Metabolomics has been used to explore links between phenotypes and metabolism. Metabolites represent intermediate and end-products of cellular regulatory processes, and their levels can reflect physiologic and pathologic changes that may mirror the progression of diseases. Thus, they may reflect metabolic changes at early stages of a disease. Therefore, metabolomics is a useful method to deepen the understanding of disease-relevant metabolic processes and dysregulation, by detecting alterations of the metabolic profile and specific metabolites.

Metabolic perturbations of prediabetes and diabetes are complex. Cross-sectional analyses have reported associations of altered metabolite levels with obesity (4), insulin resistance (5), prediabetes (6), and overt diabetes (7). Previous findings suggested that branched-chain amino acids (BCAAs) were associated with insulin resistance (5) and T2D (7). Further, the components of nitrogen metabolism pathway were also considered as potential effectors of earliest stages of T2D pathophysiology (8). Although metabolomics studies of diabetes and prediabetes are increasing in number and attracting the interest of the medical community, a clear understanding of alterations in metabolite profiles during the progression from prediabetes to diabetes has not been achieved. Identification of these changes may be useful for estimating the risk of developing prediabetes and diabetes.

Thus, the purpose of this study was to identify new metabolic markers that may help understand the pathogenesis from prediabetes to T2D, and improve risk prediction for the development of diabetes. Patients with prediabetes diagnosed by oral glucose tolerance test (OGTT) who had developed diabetes after a follow-up of 5 years were enrolled. Matched controls were also randomly selected from participants who were diagnosed with prediabetes at baseline and after a follow-up period of 5 years. We assessed and compared metabolite levels in both groups to determine which were predictive of conversion from prediabetes to diabetes, and to investigate correlations between metabolites and clinical indexes during the development of diabetes. In addition, we also investigated novel metabolites associated with risk of developing T2D.

## Research Design and Methods

### Study Population

The study population was from the Risk Evaluation of Cancers in Chinese Diabetic Individuals: A Longitudinal (REACTION) study, which was a multicenter prospective observational study aiming to evaluate chronic diseases among the Chinese population (9). The Institutional Review Boards at each study site approved the study protocol, and all participants provided written informed consent. During the recruitment period, local permanent residents (39–79 years of age) were invited to participate in a screening examination for diabetes. From June to December 2011, 3,620 residents were successfully recruited, among whom 1,256 participants were diagnosed with prediabetes. In 2016, participants returned for a 5-year follow-up survey. We randomly selected 153 individuals who reported a diagnosis of prediabetes at baseline (2011) and turned to T2D after a 5-year follow-up (2016). They did not receive medication interventions. Matched controls (n = 160) were randomly selected from participants who were diagnosed with prediabetes at both baseline and after a 5-year follow-up. The controls were matched (1.04:1) for sex, age (± 3 years), BMI and date of blood collection (± 6 months), because these factors are well-known to influence metabolic profiles (10, 11). Diabetes was defined according to the American Diabetes Association (ADA) 2010 criteria. The diagnosis of prediabetes was defined as a fasting plasma glucose (Glu0) level of 5.6-6.9 mmol/L (100-125 mg/dl), a 2-h blood glucose (Glu120) level of 7.8-11.0 mmol/L (140-199 mg/dl), or a glycated hemoglobin (HbA1c) level of 5.7- 6.4% (39-46 mmol/mol), according to the 2010 ADA guidelines (12). Serum samples were all collected by Sun Yat-Sen Memorial Hospital, of Sun Yat-Sen University, and stored at -80°C until testing.

### Clinical and Biochemical Measurements

We collected information regarding lifestyle factors, medical history, sociodemographic characteristics, and family history using a standardized questionnaire (9). Anthropometrical measurements of all participants were obtained by trained staff using standard protocols (13). Blood pressure (BP) was measured 3 times, consecutively, by the same observer within 5 minutes using an automated electronic device (OMRON, Omron Company, China). Body height and body weight were recorded to the nearest 0.1 cm and 0.1 kg, respectively, with participants were wearing light indoor clothing without shoes. Body mass index (BMI) was calculated as weight in kilograms divided by height in meters squared (kg/m^2^). Waist circumference (WC) was measured at the umbilical level with participants in the standing position at the end of a gentle expiration. All participants received a standard 75-g oral glucose tolerance test (OGTT), and plasma glucose concentrations were determined at Glu0 and Glu120. Venous blood samples were collected after an overnight fast of at least 10 h. Glu0, Glu120, triglyceride (TG), total cholesterol (TC), high-density lipoprotein cholesterol (HDL-C), low-density lipoprotein cholesterol (LDL-C), creatinine (Cr), g-glutamyltransferase (GGT), aspartate aminotransferase (AST), and alanine aminotransferase (ALT) concentrations were measured with an autoanalyzer (CX-7 Biochemical Autoanalyser; Beckman, Brea, CA, USA). HbA1c was measured by high-performance liquid chromatography (HPLC) (BioRad, Hercules, CA). Triglyceride-glucose index (TyG index) was calculated as the LN [fasting TG(mg/dl)*Glu0(mg/dl)/2] (14). The first morning spot urine samples were collected for assessing the urine albumin (Urine-ALB).

### Serum Collection and Preparation

Antecubital venous blood samples (20 ml) were obtained after a 10 h fast, and samples were immediately placed on ice. Samples were processed within 6 h to obtain serum, which was stored at -80°C until testing. For metabolic profiling, serum samples were thawed on ice and metabolites were extracted with methanol using a previously described method (15). A total of 50 μl of thawed serum was collected and precipitated by 150 μl of methanol with 10 μl of 1 mg/ml 2-chloro-L-phenylalanine as the internal standard. After centrifugation at 14,000 ×*g* for 10 min at 4°C, the supernatant was transferred to a 1.5 ml sample vial. A pooled quality control (QC) sample was prepared by mixing equal amounts (10 μl) of each serum sample.

### Serum Metabolomics Profiling by LC-Mass Spectrometry (LC-MS)

The prepared samples were analyzed using an ultraperformance HPLC (UHPLC) system (1290, Agilent Technologies) with a UPLC HSS T3 column (2.1 mm × 100 mm, 1.8 μm, Waters) coupled to Q Exactive Focus (Thermo Fisher Scientific, MA, USA), *via* a previously described method with some modifications (16). Additional details are provided in the **Supplementary Material Methods** section.

### Data Analysis

Metabolite levels were log-transformed for analysis. To compare metabolite levels between the prediabetes and T2D groups, a paired Mann-Whitney-Wilcoxon test (a nonparametric univariate method), and multivariate statistical analysis [PCA (Principal Component Analysis) and OPLS-DA (Orthogonal Partial Least Squares Discriminant Analysis)] were used, and analyses were conducted with an R software (*v* 4.1.0) and SIMCA 15.0 software (Umetrics, Umea, Sweden). Metabolites that were found to be significant, were adjusted for confounding factors (Age, BMI, WC, waist-hip ratio, urine albumin, HDL-C, LDL-C, TC, GGT, Glu0, Glu120, HbA1C) and examined by multivariate logistic regression analysis to determine their value for discriminating prediabetes from T2D.

Metabolomics Pathway Analysis (MetPA, v5.0) was performed to identify significantly altered pathways contributing to the progression of prediabetes to diabetes. Permutation multivariate analysis of variance (PERMANOVA) (Bray–Curtis distance) was employed to test statistically significant differences between metabolic profiles and individuals’ phenotypes (17). Multivariate logistic regression analysis was performed with adjustment for confounding factors to estimate the association between each novel metabolite and diabetes risk. An adjusted P-value corrected for multiple tests using a false discovery rate (FDR) (Benjamini-Hochberg) of < 0.05 was regarded as significant.

Next, the random forest classifier (RFC) for discriminating prediabetes from T2D was trained on 182 randomly selected subjects (91 with prediabetes, 91 with diabetes) from the 306 participants, and then tested on the remaining subjects (62 with prediabetes, 62 with diabetes). The analysis was conducted with 5 repetitions of 10-fold cross-validation, using cross-validation error curves to select features as described by Feng et al. (18). The cross-validation error curves from the 5 trials were averaged, and the minimum error in the averaged curve plus the standard deviation at that point was used as the cutoff for an acceptable error. From the sets of metabolites with a classification error less than the cutoff, the set with the smallest number of metabolites was chosen as the optimal set (19). The risk probability of diabetes for each subject was computed, and the area under the receiver operating characteristics (ROC) curve (AUC) was calculated. The RFC model was further tested and validated on another dataset (160 prediabetes subjects who were still in prediabetes status in the 5-year follow-up). Clinical indexes were also applied for RFC construction. Furthermore, the combinations of the optimal metabolites and clinical factors sets were applied for RFC model to compare diabetes prediction capability.

## Results

The characteristics of pre-diabetes and matched control at baseline were shown in **Table 1**. Overall, despite of no differences in serum cholesterol and liver functions, significantly higher baseline Glu120, HbA1c and urine albumin levels were found in the pre-diabetes group who turned to diabetes than those who were still in pre-diabetes status after the 5-year of follow-up.

**Table 1:** The characteristics of Pre-diabetes and matched control at baseline.

Variables	Pre-Diabetes Group# (Baseline )	Pre-Diabetes Matched Control Group (Baseline )	p.value*
Age	56 ± 7	56 ± 7	0.79
Gender (male/female)	49/104	46/114	0.61
BMI	24.2 ± 2.8	24.4 ± 2.7	0.50
SBP	129 ± 14	129 ± 14	0.87
DBP	76 ± 9	78 ± 10	0.16
WC	83 ± 8	84 ± 8	0.76
HC	95 ± 7	96 ± 6	0.24
Waist-hip ratio	0.88 ± 0.06	0.87 ± 0.06	0.44
HR	82 ± 11	81 ± 11	0.98
HDL-C (mmol/L)	1.22 ± 0.36	1.24 ± 0.33	0.97
LDL-C (mmol/L)	3.11 ± 0.94	3.11 ± 0.92	0.81
TC (mmol/L)	5.21 ± 1.26	5.16 ± 1.23	0.50
TG (mmol/L)	2.06 ± 1.90	1.84 ± 1.26	0.73
ALT (U/L)	16 ± 9	16 ± 10	0.70
AST (U/L)	19 ± 7	19 ± 7	0.50
GGT (mg/dL)	28 ± 18	26 ± 21	0.11
Glu0 (mmol/L)	5.75 ± 0.64	5.63 ± 0.61	0.09
Glu120 (mmol/L)	9.19 ± 1.16	8.73 ± 1.21	<0.001
HbA1c (%)	5.97 ± 0.35	5.89 ± 0.35	0.02
Urine-ALB	8.90 ± 10.39	7.81 ± 9.39	0.03

# mean ± SD or number of individuals (%). *P. value was calculated by the two-tailed Wilcoxon rank-sum tests (continuous variables) or chi-square tests (discontinuous variables). BMI, Body Mass Index; SBP, Systolic blood pressure; DBP, Diastolic blood pressure; WC, Waist circumference; HC, hip circumference; HR, Heart Rate; HDL-C, High-density lipoprotein cholesterol; LDL-C, Low-density lipoprotein cholesterol; TC, Total cholesterol; TG, Triglyceride; ALT, Alanine aminotransferase; AST, Aspartate aminotransferase; GGT, G-glutamyltransferase; Glu0, fasting plasma glucose; Glu120, 2-h blood glucose. Urine-ALB, urine albumin.

The characteristics of pre-diabetes and matched control after 5-year follow-up are shown in **Supplementary Table 1**. The mean Glu0 and Glu120 values in prediabetes group at baseline were 5.75 ± 0.64 mmol/L and 9.19 ± 1.16 mmol/L, respectively, and they turned to diabetes after 5-year follow-up with the values as 6.27 ± 1.36 mmol/L and 12.90 ± 2.84 mmol/L, respectively. Besides, patients who developed diabetes after the 5-year of follow-up had higher levels of BMI, WC, waist-hip ratio as well as HbA1c comparing with prediabetes group. A comparison of diabetes risk factors between the groups showed that individuals with diabetes were more likely to be hypercholesterolemic compared with individually matched controls, and had significantly higher LDL-C, TC, and urine albumin levels. Levels of GGT were significantly different between the 2 groups, but the mean value of both groups was within normal limits.

**Figure 1:**
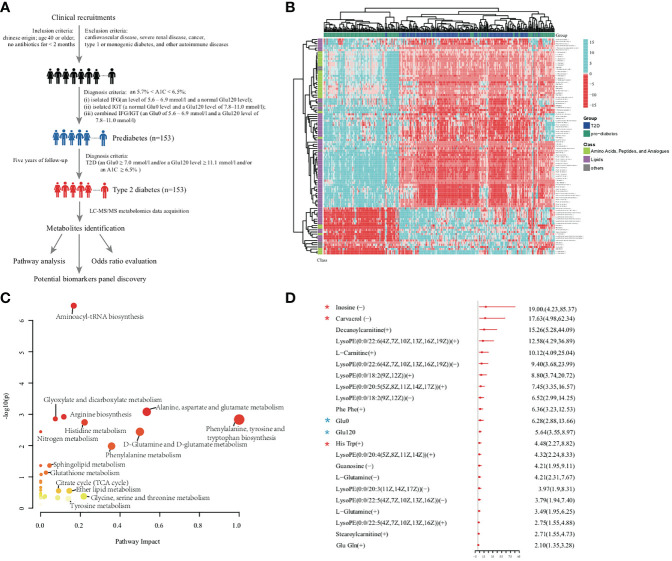
**(A)** Overview of workflow in this study. **(B)** Heatmap of 101 significant metabolites. Amino acid and lipids showed obvious differences between prediabetes group and diabetes group from the results of metabolites abundance heatmap. **(C)** Metabolomic pathway analysis highlighted the potential importance of distinct pathways that were represented by metabolites associated with diabetes. Each bubble present one pathway. The color and size of each circle was based on P values and pathway impact values, respectively. **(D)** The OR per standard deviation increment and 95% CI estimation for the association between each novel metabolite and increased diabetes risk. ORs and 95% CIs of diabetes for the comparison between highest versus lowest tertile of clinical parameters and metabolites were adjusted for confounding factors (BMI, Age, WC, Waist–hip ratio, urine ALB, HDL-C, LDL-C, TC, GGT, Glu0, Glu120, HbA1_C_) discriminating prediabetes group from diabetes group by multi-logistic regression analysis. Red star indicated selected biomarkers by RFC model, blue stars indicated conventional diabetes diagnosis factors.

**Figure 1A** provides a detailed workflow of the metabolomics study. The metabolomics data acquired resulted in 3,531 positive mode peaks and 4,849 negative mode peaks after quality control. No significant drifts in the QC (quality control) metabolites profiles obtained in both ion modes were observed, and the profiles demonstrated relatively good stability and reproducibility. Both PCA scores plot and OPLS-DA scores plot, validated with permutation tests (200 permutations) (20), revealed significant metabolite differences between the prediabetes group and the diabetes group. PERMANOVA analysis demonstrated diabetes status exhibited significant impact on the metabolic profile (Q-value < 0.05, 999 permutations). Overall, the serum metabolome datasets were significantly associated with Glu120 (Q-value < 0.05) (**Supplementary Table 2**). Weak interactions were observed between the global metabolomics profiles and clinical parameters such as LDL-C and TC (P-value < 0.05, Q-value > 0.05). Furthermore, age and Glu0 were weakly associated with serum metabolome profile in women, and smoking behavior was weakly associated with serum metabolites profile in men (20).

The 22 significantly up-regulated and 79 significantly down-regulated metabolites independent of conventional risk factors in the T2D group are shown in **Figure 1B**; the metabolites were mainly components of amino acid metabolism and lipid metabolism (20). MetPA analysis indicated that notable metabolism dysregulation occurred in the progression of T2D. As shown in **Figure 1C**, amino acids metabolism and lipids metabolism were statistically different between the prediabetes and diabetes groups. This was especially apparent with respect to Aminoacyl-tRNA biosynthesis, Alanine, aspartate and glutamate metabolism, Phenylalanine, tyrosine and tryptophan biosynthesis, and Sphingolipid metabolism (20).

The odds ratio (OR) associated with metabolites and clinical factors for the risk of diabetes after adjustment for confounding factors are summarized in **Figure 1D**. Multivariable logistic regression analyses revealed 20 altered metabolites were significantly associated with an increased risk of diabetes after adjustment for clinical factors and FDR correction. In the multivariate model, the OR of inosine for developing diabetes was 19.00 (95% confidence interval [CI]: 4.23-85.37), and the OR of carvacrol was 17.63 (95% CI: 4.98-62.34). Glu0 was independently associated with development of diabetes (OR = 6.28; 95% CI: 2.88-13.66), as was Glu120 level (OR = 5.64; 95% CI: 3.55-8.97). These diabetes positively associated metabolites such as inosine, carvacrol, and decan oylcarnitine might be complementary to glucose in improving diabetes forewarning, which would be further validated in the future study.

**Figure 2:**
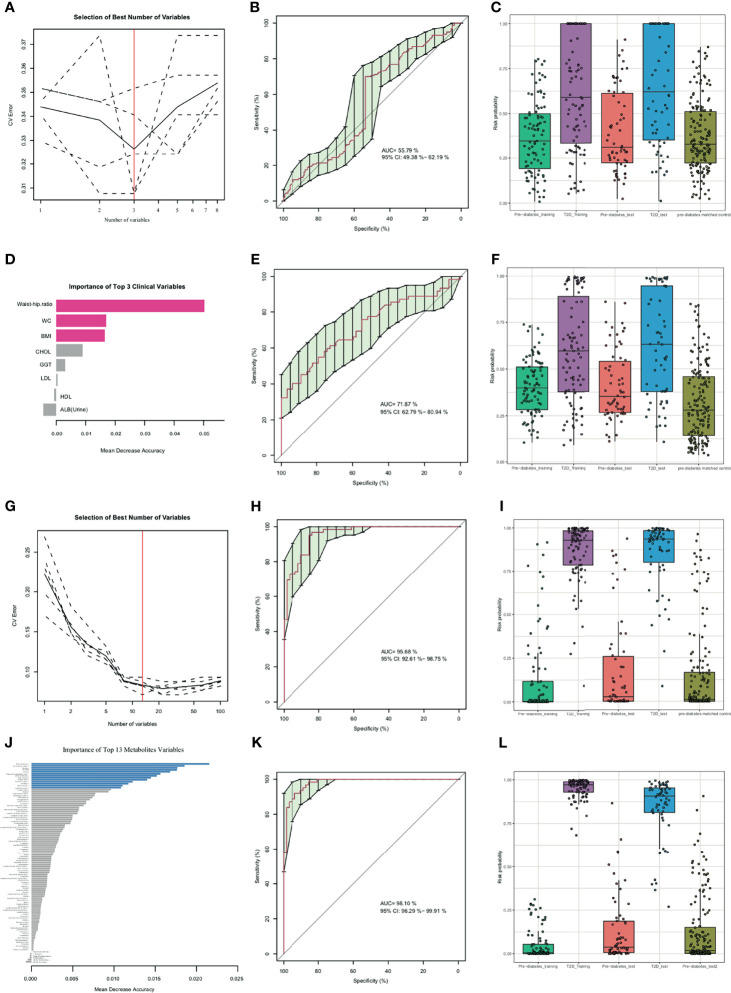
Random forest classification (RFC) model based on clinical parameters **(A-F)**, metabolites **(G-J)** and potential biomarkers panel discovery and evaluation **(K, L)**. **(A, G)** Distribution of 5 trials of 10-fold cross-validation error in random forest classifiers. The model was trained with clinical factors in the training set (prediabetes group, n=91; diabetes group, n=91). The black solid curve showed the average of the 5 trials (dash lines). The red line indicated the number of picked features in the optimal set. **(B, E, H, K)** Receiver Operating Characteristic curve (ROC curve) and area under the ROC curve of 3 selected clinical indexes **(B)**, 3 selected clinical indexes and 5 conventional prediction indexes (DBP, SBP, TG, LDL and TyG index) **(E)**, 13 selected metabolites **(H)**, 13 selected metabolites and 8 clinical indexes **(K)** for the test set with prediabetes subjects (n=62) and diabetes subjects (n=62). **(C, F, I, L)** Box-and-whisker plot presents the risk probability of developing diabetes among the training datasets (prediabetes group, n=91; diabetes group, n=91), validation datasets 1 (prediabetes group, n=62; diabetes group, n=62), validation datasets 2 (pre-diabetes matched control, n=160) according to the RFC model, Age was additionally added to predict validation datasets 2. **(D, J)** The importance of clinical variables and metabolites variables. The red color indicated selected clinical indexes and the blue color indicated selected optimal metabolites panel.

A RFC was established to investigate whether metabolic profiling or clinic profiling could predict future diabetes development in prediabetic subjects independent of primary diagnostic criteria of diabetes (Glu0, Glu120, and HbA1C). As age variant was a conventional diabetes risk factor, it was additionally added in the performance evaluation of the diabetes risk model in the independent dataset (pre-diabetes matched control) in the after-mentioned RFC model. As shown in **Figures 2A–D**, the RFC risk model contained 3 clinical factors (BMI, waist-hip ratio, and WC), and the validated AUC for diabetes development prediction was 55.79% (95% CI: 49.38-62.19%). Recent studies have shown TyG index in comparison with fasting plasma glucose improved diabetes prediction in patients with normal fasting glucose (14), so it was also analyzed as a conventional risk index. To further explore whether other 5 conventional risk indexes (such as DBP, SBP, TG, LDL and TyG index) could improve the prediction capability of diabetes progress, and new RFC model with aforementioned 8 clinical indexes (BMI, waist-hip ratio, WC, DBP, SBP, TG, LDL and TyG index) was established which showed a better prediction performance with AUC of 71.87% (62.79%-80.94%) (**Figures 2E, F**).

The predictive performance of metabolites for diabetes development prediction was also examined using the same numbers of random selected subjects as the training and validation datasets. As illustrated in **Figures 2G–J** and **Table 2**, a model containing 13 features was successfully generated; the model exhibited an AUC of 95.68% (95% CI: 92.61-98.75%) for diabetes development prediction (**Figure 2H**). The risk probability of all subjects was estimated, and subjects with diabetes and prediabetes in the validation sets could be more broadly separated than clinical indexes (**Figure 2I**).

**Table 2:** 13 features discriminating diabetes patients from prediabetes subjects selected with Random Forest model.

Name	mz	RT(min)*	ppm	Ion	FORMULA	Super_class	VIP§	Foldchange (T2D/ Prediabetes)	P value	q value‡
Inosine(-)	267.07	2.26	0.56	H-	C10H12N4O5	Nucleosides, Nucleotides, and Analogues	2.36	4.29	6.44E-23	3.89E-22
PC(P-17:0/0:0)(+)	494.36	7.54	3.59	H+	C25H52NO6P	Lipids	3.03	0.13	7.98E-25	1.42E-23
PC(O-16:0/3:1(2E))(+)	536.37	7.49	3.75	H+	C27H54NO7P	Lipids	1.86	0.48	5.60E-24	5.27E-23
Carvacrol(-)	149.10	5.95	0.88	H-	C10H14O	others	1.33	1.66	8.09E-23	4.70E-22
PC(O-16:0/O-1:0)(+)	496.37	7.57	3.11	H+	C25H54NO6P	Lipids	3.19	0.11	8.43E-25	1.46E-23
PC(O-18:0/O-2:1(1E))(+)	536.41	8.22	3.85	H+	C28H58NO6P	Lipids	3.21	0.10	7.40E-26	6.53E-24
LysoPC(20:1(11Z))(+)	550.38	7.87	3.44	H+	C28H56NO7P	Lipids	1.79	0.43	1.12E-25	7.07E-24
Phe Phe(+)	313.15	3.72	3.30	H+	C18H20N2O3	Amino Acids, Peptides, and Analogues	2.80	3.03	3.30E-21	1.24E-20
PE(P-16:0e/0:0)(-)	436.28	7.11	0.13	H-	C21H44NO6P	Lipids	2.36	0.22	3.37E-24	3.58E-23
PE(O-18:1(9Z)/0:0)(-)	464.31	7.84	0.14	H-	C23H48NO6P	Lipids	2.36	0.22	2.97E-24	3.27E-23
LysoPC(P-16:0)(+)	480.34	7.17	3.26	H+	C24H50NO6P	Lipids	2.31	0.24	7.40E-26	6.53E-24
1-Palmitoyl Lysophosphatidic Acid(-)	409.24	6.07	0.02	H-	C19H39O7P	Lipids	2.57	0.15	7.14E-25	1.34E-23
L-Histidine(-)	154.06	0.58	0.27	H-	C6H9N3O2	Amino Acids, Peptides, and Analogues	1.62	0.41	1.42E-22	7.56E-22

*Retention time. §VIP (Variable Importance for Projection),one indicator reflecting the capability of the variables to explain Y. ‡Adjusted p.value calculated by the paired two-tailed Wilcoxon rank-sum tests after false discovery rate correction.

To investigate whether the identified metabolites improved diabetes risk prediction, the same numbers of random selected subjects as the training and validation sets were used to construct a risk prediction model of diabetes by combination of the 8 clinical indexes, and 13 metabolites selected above. The AUC of the new combinational model was 98.10% (95% CI: 96.29-99.91%) (**Figure 2K**). As shown in **Figure 2L**, the combination risk prediction model provided an increased predictive value comparing to the metabolites panel alone, or the clinical features model alone. Together, these results indicated that the metabolome profile was regulated in a complex manner during development from prediabetes to diabetes.

## Discussion

In this prospective investigation using an untargeted high-resolution metabolomics approach, we detected alterations in serum metabolites preceding the onset of diabetes from prediabetes by about 5 years. In addition, we identified metabolites that were associated with risk of diabetes, and improved the ability to predict the development of diabetes beyond that using routine clinical risk factors. Prior metabolomics studies have focused on a mixture of individuals with normoglycemia and prevalent dysglycemia (21–23), whereas the current study is novel in the respect that the participants had baseline prediabetes and turned to T2D.

To identify metabolites that were altered between participants with prediabetes and those with T2D, we performed non-targeted serum metabolomics profiling of baseline prediabetes and follow-up T2D groups in order to reduce noise due to inter-individual variability. The significant metabolic signatures identified in this study can be broadly classified into those associated with amino acid metabolism, especially aromatic amino acids, and lipid metabolism. Prior studies have reported increased circulating levels of branch-chain amino acids (BCAAs) and aromatic amino acids are associated with insulin resistance (2, 24) and diabetes (25, 26). In the current study, the amino acids phenylalanine and tyrosine were down-regulated in diabetes group compared with the prediabetes group. Besides, the BCAA valine (VIP value=0.78, Q-value=5.04E-18, fold-change (diabetes/prediabetes) =1.26) was up-regulated and isoleucine (VIP value=0.29, Q-value=0.001, fold-change (diabetes/prediabetes) =0.94) was down-regulated in diabetes group based on the univariate statistics. On the other hand, phenylalanylphenylalanine, which is reported to be positively associated with pancreatic ductal adenocarcinoma (27), was significantly up-regulated in diabetes group and associated with an increased risk of diabetes.

In insulin-resistant states, the body aims to compensate for decreased peripheral tissue glucose uptake through increased pancreatic insulin secretion (28). Phenylalanine, which is positively associated with insulin secretion, may be involved compensation pathways in the early stages of insulin resistance *via* the stimulation of insulin secretion. Once the ability for compensated insulin secretion is reached, there is progression to overt diabetes. Our findings of early metabolite changes in the progression of prediabetes into diabetes are consistent with a recent Mendelian randomization analysis, that reported that elevations in amino acid levels occur after the development of insulin resistance (29). Aromatic amino acids may be potential markers for dysglycemic states; the precise role and function of the amino acids with respect to diabetes have yet to be identified. Although previous observational studies have reported associations of circulating aromatic amino acids with adverse events in non-diabetic persons, the present report for the first time has identified increased circulating levels of certain amino acids are associated with an increased risk of prediabetes progressing to diabetes. Interestingly, other metabolites such as BCCAs which were associated with diabetes incidence in previous studies were not prioritized by our selection algorithm; however, this finding is consistent with the results of a metabolomics study of American Indians (30). It is possible that the unique characteristics of the Chinese population, e.g., genetic background and lifestyle, could result in a unique population-specific metabolomics signature.

In this study we identified 13 metabolites, 3 up-regulated and 10 down-regulated, that independently predicted the progression from prediabetes to T2D. It is noteworthy that inosine, carvacrol, and carnitine were all associated with increased risk of developing diabetes from prediabetes, and the associations were independent of classical risk factors. Inosine, a naturally occurring purine, was long considered to be an inactive metabolite of adenosine. However, recent studies have shown that inosine has immunomodulatory and anti-inflammatory properties *in vitro* and *in vivo* (31). Inosine is a potent stimulator of insulin secretion from isolated mouse islet cells (32), but its relation with diabetes development in humans remains unknown. Carvacrol is a predominant constituent of essential oils and has well-known antioxidant, antimicrobial, antifungal, and anti-inflammatory properties (33, 34), and exerted an anti-hyperglycemic effect in STZ-induced diabetic mice and a protective role in diabetes-induced aortic hypercontractility (35, 36). In the present study, the OR associated with metabolites and clinical factors for the risk of diabetes after adjustment for confounding factors are analyzed. The OR of carvacrol was 17.63 (95% CI: 4.98-62.34), which was considered as complementary to glucose in improving diabetes forewarning, However, the role of carvacrol in the progression from prediabetes to diabetes in human remained unknown. Amino acid derivative carnitine is primarily synthesized in the liver in its L-form from lysine and methionine, and transported *via* the bloodstream to cardiac and skeletal muscle. It is required for mitochondrial fatty acid β-oxidation and the transport of long-chain fatty acids across the inner membrane of the mitochondria, in the form of acyl-carnitine, where they can be metabolized for energy. Carnitine has been used in a number of human studies alone or as part of a combination therapy (37, 38). However, a recent study indicated that dietary L-carnitine could be converted into the atherosclerosis- and thrombosis-promoting metabolite trimethylamine N-oxide *via* sequential gut microbiota–dependent transformations (39). While the 3 metabolites have been well-studied, the mechanisms by which the affect diabetes risk is not clear. Maybe the increased metabolites that occurs at the stage of diabetes is not part of the pathogenic mechanism that induces or maintains the disease. They only represent a kind of protective mechanism associated with recovery from the abnormal glucose metabolism in body. Future investigations are warranted to better understand the effects of these metabolites, and their relations with diabetes risk.

An important finding of this study is that the combination of the identified metabolites can predict the development of T2D from prediabetes better than using conventional risk factors. The AUC of 8 conventional clinical risk factors for predicting the development of T2D from prediabetes was only 0.72, which is lower than reported in other recent studies of normoglycemic individuals (40). A possible explanation might be related to the study population: in persons with prediabetes the predictive value of traditional T2D risk factors may not be as strong as in persons who are normoglycemic. A model that combined 13 metabolomics signatures and 8 traditional risk factors exhibited an AUC of 0.98 for predicting the development of diabetes from prediabetes. It is noteworthy that we observed significant associations between long chain-phospholipids and diabetes risk; long chain-phospholipids were found to be predictive of diabetes in the prospective Framingham Offspring cohort and the Malmö Diet and Cancer study (24). Similar observations have recently been reported with respect to the fatty acid compositions of triglycerides (41), suggesting that lipids with a shorter chain length may trigger development of diabetes, whereas those containing longer chains may offer protection.

In comparing of persons with prediabetes and those with T2D, we observed a significant reduction of lysophosphatidylcholine (LysoPC) species in diabetes patients, including LysoPC (20:1(11Z)) and LysoPC (P-16:0), which were selected by the diabetes prediction model. LysoPC is an important signaling molecule and fatty acid carrier, and accounts for 5–20% of total plasma phospholipids. Alterations in LysoPC species have been widely studied in relation to diabetes and obesity. A large cross-sectional study reported significantly lower levels of several LysoPC species in patients with impaired glucose tolerance and diabetes (42). *In vivo* and *in vitro* studies have reported that LysoPC species enhance glucose-dependent insulin secretion *via* G-protein-coupled receptor G119 (43).

There are some limitations to this study that should be considered. First, because all of the participants were Chinese, the conclusions may not be generalizable to other ethnicities. Second, it is possible that certain unstable metabolites will degrade during sample collection, storage, and processing; however, all samples were collected according to a standardized protocol and stored at -80°C. Plasma metabolites detected by UPLC-QTOF-MS have been shown to be stable over a period of 13-17 years when stored at -80°C. Thus, we do not believe that sample handling or storage had any marked effect on the study results. Third, we acknowledge that the results of our population-based analysis should be interpreted with caution since some unmeasured factors (e.g. changes in lifestyle factors, pre-clinical treatments or other diseased states over time) might have influenced our findings. Lastly, insulin level data were not available; thus, insulin resistance and insulin sensitivity could not be evaluated. In recent studies, TyG index was identified as a risk marker of insulin resistance, so we used TyG index as a conventional factor for diabetes prediction in prediabetes patients (44).

In conclusion, this study identified a discrete set of metabolites associated with an increased risk of prediabetic persons developing T2D. Changes in these metabolites preceded the onset of overt diabetes, and in clinical practice may help to identify individuals with prediabetes who are at increased risk of progressing to overt diabetes.

## Data Availability Statement

The datasets presented in this study can be found in online repositories. The names of the repository/repositories and accession number(s) can be found in the article/**Supplementary Material**.

## Ethics Statement

The studies involving human participants were reviewed and approved by Sun Yat-Sen Memorial Hospital, of Sun Yat-Sen University. The patients/participants provided their written informed consent to participate in this study. Written informed consent was obtained from the individual(s) for the publication of any potentially identifiable images or data included in this article.

## Author Contributions

MR researched the literature, designed the experiments, analyzed the data, interpreted the results, and wrote and approved the manuscript. DL and KS performed the collection of clinical data and the statistical analysis. ZL, YQ, and JD interpreted the results, analyzed the data, and collaborated with all other authors. GB conducted and supervised the metabolomics measurements. XW and GJ also collected the clinical data. JL, JH, and CW analyzed data and drafted the manuscript. LY and MR are the guarantors of this work and, as such, have full access to all the data in the study and take responsibility for the integrity of the data and the accuracy of the data analysis. All authors contributed to the article and approved the submitted version.

## Funding

This work was supported by grants from the National Natural Science Foundation of China (81870571,81770827).

## Conflict of Interest

The authors declare that the research was conducted in the absence of any commercial or financial relationships that could be construed as a potential conflict of interest.

## Publisher’s Note

All claims expressed in this article are solely those of the authors and do not necessarily represent those of their affiliated organizations, or those of the publisher, the editors and the reviewers. Any product that may be evaluated in this article, or claim that may be made by its manufacturer, is not guaranteed or endorsed by the publisher.
