# Supplementary material for: Potential Novel Serum Metabolic Markers Associated With Progression of Prediabetes to Overt Diabetes in a Chinese Population
Source: Front Endocrinol (Lausanne). 2022 Jan 5;12:745214. doi: 10.3389/fendo.2021.745214 (PMC8766640; doi:10.3389/fendo.2021.745214)
Supplement: Supplementary file 1 [file DataSheet_1.docx]

**Supplementary Table S1. The characteristics of Pre-diabetes and matched control at baseline and after 5 year follow-up**

| **Variables** | **Pre-diabetes group# (baseline )** | **T2D**  **(5-year follow-up of pre-diabetes group )** | **Pre-diabetes matched control group (baseline )** | **Pre-diabetes matched control after 5-year follow-up** | **p.value*** | | | |
| --- | --- | --- | --- | --- | --- | --- | --- | --- |
|  |  |  |  |  | **Pre-diabetes vs T2D** | **Pre-diabetes vs Pre-diabetes matched control** | **Pre-diabetes matched control**  **（baseline vs. 5 year follow up）** | **T2D vs control after 5-year follow-up** |
| Age | 56±7 | 61±7 | 56±7 | 61±7 | <0.001 | 0.79 | <0.001 | 0.77 |
| Gender(male/female) | 49/104 | 49/104 | 46/114 | 46/104 | 1.00 | 0.61 | 1 | 0.61 |
| BMI | 24.2±2.8 | 24.5±3.1 | 24.4±2.7 | 24.2±3.0 | 0.03 | 0.50 | 0.42 | 0.71 |
| SBP | 129±14 | 129±15 | 129±14 | 127±13 | 0.93 | 0.87 | 0.27 | 0.27 |
| DBP | 76±9 | 76±9 | 78±10 | 76±9 | 0.12 | 0.16 | 0.32 | 0.99 |
| WC | 83±8 | 87±10 | 84±8 | 86±9 | <0.001 | 0.76 | 0.04 | 0.83 |
| HC | 95±7 | 96±9 | 96±6 | 97±9 | 0.12 | 0.24 | 0.70 | 0.16 |
| Waist-hip ratio | 0.88±0.06 | 0.91±0.07 | 0.87±0.06 | 0.88±0.08 | <0.001 | 0.44 | 0.07 | 0.06 |
| HR | 82±11 | 80±10 | 81±11 | 79±11 | 0.13 | 0.98 | 0.14 | 0.34 |
| HDL-C(mmol/L) | 1.22±0.36 | 1.32±0.30 | 1.24±0.33 | 1.34±0.32 | <0.001 | 0.97 | 0.01 | 0.92 |
| LDL-C(mmol/L) | 3.11±0.94 | 3.56±0.95 | 3.11±0.92 | 3.54±0.97 | <0.001 | 0.81 | <0.001 | 0.77 |
| TC(mmol/L) | 5.21±1.26 | 5.98±1.31 | 5.16±1.23 | 5.88±1.12 | <0.001 | 0.50 | <0.001 | 0.76 |
| TG(mmol/L) | 2.06±1.90 | 2.13±1.61 | 1.84±1.26 | 1.91±1.32 | 0.07 | 0.73 | 0.82 | 0.05 |
| ALT(U/L) | 16±9 | 16±14 | 16±10 | 14±23 | 0.75 | 0.70 | 0.01 | 0.03 |
| AST(U/L) | 19±7 | 21±8 | 19±7 | 19±8 | 0.18 | 0.50 | 0.73 | 0.02 |
| GGT(mg/dL) | 28±18 | 37±31 | 26±21 | 30±25 | <0.001 | 0.11 | 0.02 | <0.001 |
| Glu0(mmol/L) | 5.75±0.64 | 6.27±1.36 | 5.63±0.61 | 5.48±0.51 | <0.001 | 0.09 | 0.04 | <0.001 |
| Glu120(mmol/L) | 9.19±1.16 | 12.90±2.84 | 8.73±1.21 | 9.00±1.06 | <0.001 | <0.001 | 0.08 | <0.001 |
| HbA1c(%) | 5.97±0.35 | 6.15±0.68 | 5.89±0.35 | 5.74±0.38 | 0.04 | 0.02 | <0.001 | <0.001 |
| Urine-ALB | 8.90±10.39 | 15.37±36.18 | 7.81±9.39 | 9.34±11.35 | 0.02 | 0.03 | 0.12 | 0.02 |
| # mean ± SD or number of individuals (%)  **P*. value was calculated by the two-tailed Wilcoxon rank-sum tests (continuous variables, Pre-diabetes vs T2D and Pre-diabetes matched control baseline vs 5 year follow up with paired and others with unpaired) or chi-square tests (discontinuous variables). | | | | | | | | |

BMI, Body Mass Index; SBP, Systolic blood pressure; DBP, Diastolic blood pressure; WC, Waist circumference; HC, hip circumference; HR, Heart Rate; HDL-C, High-density lipoprotein cholesterol;

LDL-C, Low-density lipoprotein cholesterol; TC, Total cholesterol; TG, Triglyceride; ALT, Alanine aminotransferase; AST, Aspartate aminotransferase; GGT, G-glutamyltransferase;

Glu0, fasting plasma glucose; Glu120, 2-h blood glucose. Urine-ALB, urine albumin.
