# Supplementary material for: Potential Novel Serum Metabolic Markers Associated With Progression of Prediabetes to Overt Diabetes in a Chinese Population
Source: Front Endocrinol (Lausanne). 2022 Jan 5;12:745214. doi: 10.3389/fendo.2021.745214 (PMC8766640; doi:10.3389/fendo.2021.745214)
Supplement: Supplementary file 2 [file DataSheet_2.docx]

**Supplementary Table S2. PERMANOVA for the influence of phenotypes on the metabolome (999 permutations, Bray-Curtis distance).**

| **Phenotype** | **Classification** | **Prediabetes *VS.* Diabetes** | | | **Prediabetes *VS*. Diabetes (female)** | | | **Prediabetes *VS*. Diabetes**  **(male)** | | |
| --- | --- | --- | --- | --- | --- | --- | --- | --- | --- | --- |
|  |  | **Bray-Curtis distance** | | | **Bray-Curtis distance** | | | **Bray-Curtis distance** | | |
|  |  | **R^2^** | P-value | Q-value* | **R^2^** | **P-value** | **Q-value*** | **R^2^** | **P-value** | **Q-value*** |
| Group | Sample class | 0.0632 | 0.0010 | 0.0340 | 0.0622 | 0.0010 | 0.0660 | 0.0685 | 0.0020 | 0.1100 |
| Smoked | Daily habits | 0.0036 | 0.0010 | 0.0340 | 0.0054 | 0.3180 | 0.8448 | 0.0643 | 0.0070 | 0.1925 |
| Smoking |  | 0.0021 | 0.4420 | 0.8342 | 0.0075 | 0.2010 | 0.7683 | 0.0272 | 0.0770 | 0.5238 |
| Drinked |  | 0.0011 | 0.8060 | 0.9308 | 0.0044 | 0.5900 | 0.9271 | 0.0163 | 0.3120 | 0.7794 |
| Drinking |  | 0.0052 | 0.9850 | 0.9923 | 0.0117 | 0.1300 | 0.6854 | 0.0056 | 0.8870 | 0.9856 |
| Work |  | 0.0495 | 0.2560 | 0.7253 | 0.0648 | 0.0010 | 0.0660 | 0.0695 | 0.0020 | 0.1100 |
| Walk |  | 0.0220 | 0.0010 | 0.0340 | 0.0246 | 0.0050 | 0.1320 | 0.0254 | 0.0990 | 0.5238 |
| Seat1 |  | 0.0081 | 0.0060 | 0.1632 | 0.0052 | 0.4820 | 0.9014 | 0.0043 | 0.7510 | 0.9506 |
| Seat1day |  | 0.0060 | 0.1030 | 0.5603 | 0.0021 | 0.7640 | 0.9762 | 0.0068 | 0.6810 | 0.9506 |
| Seat1hou |  | 0.0013 | 0.1340 | 0.6392 | 0.0041 | 0.6240 | 0.9528 | 0.0250 | 0.0970 | 0.5238 |
| Seat2 |  | 0.0089 | 0.9610 | 0.9901 | 0.0054 | 0.4800 | 0.9014 | 0.0285 | 0.0490 | 0.4714 |
| Seat2day |  | 0.0120 | 0.0690 | 0.5558 | 0.0037 | 0.4550 | 0.9014 | 0.0071 | 0.7000 | 0.9506 |
| Seat2hou |  | 0.0025 | 0.0760 | 0.5558 | 0.0029 | 0.8710 | 0.9762 | 0.0225 | 0.1670 | 0.6967 |
| Age | Clinical factors | 0.0062 | 0.0860 | 0.5558 | 0.0197 | 0.0050 | 0.1320 | 0.0067 | 0.6180 | 0.9506 |
| Gender |  | 0.0043 | 0.2340 | 0.6948 | NA | NA | NA | NA | NA | NA |
| BMI |  | 0.0018 | 0.8450 | 0.9308 | 0.0113 | 0.0730 | 0.5500 | 0.0134 | 0.3300 | 0.7794 |
| SBP |  | 0.0043 | 0.3150 | 0.7650 | 0.0019 | 0.9370 | 0.9762 | 0.0069 | 0.7300 | 0.9506 |
| DBP |  | 0.0055 | 0.2210 | 0.6948 | 0.0056 | 0.3820 | 0.9014 | 0.0101 | 0.5090 | 0.9460 |
| HR |  | 0.0017 | 0.8620 | 0.9308 | 0.0042 | 0.5750 | 0.9145 | 0.0064 | 0.7930 | 0.9506 |
| WC |  | 0.0082 | 0.0600 | 0.5558 | 0.0093 | 0.1350 | 0.6854 | 0.0140 | 0.2950 | 0.7794 |
| HC |  | 0.0079 | 0.0830 | 0.5558 | 0.0071 | 0.2750 | 0.8442 | 0.0159 | 0.2210 | 0.7367 |
| Waist.hip.ratio |  | 0.0032 | 0.4700 | 0.8342 | 0.0068 | 0.2960 | 0.8448 | 0.0170 | 0.2070 | 0.7367 |
| HDL-C |  | 0.0032 | 0.3910 | 0.8282 | 0.0039 | 0.5080 | 0.9014 | 0.0048 | 0.7750 | 0.9506 |
| LDL-C |  | 0.0098 | 0.0220 | 0.3400 | 0.0098 | 0.0530 | 0.4538 | 0.0210 | 0.0960 | 0.5238 |
| TC |  | 0.0110 | 0.0080 | 0.1813 | 0.0116 | 0.0540 | 0.4538 | 0.0076 | 0.5440 | 0.9506 |
| TG |  | 0.0048 | 0.1760 | 0.6800 | 0.0025 | 0.7430 | 0.9762 | 0.0048 | 0.7880 | 0.9506 |
| ALT |  | 0.0033 | 0.3820 | 0.8282 | 0.0019 | 0.8740 | 0.9762 | 0.0024 | 0.9860 | 1.0000 |
| AST |  | 0.0023 | 0.6170 | 0.8476 | 0.0025 | 0.7840 | 0.9762 | 0.0040 | 0.8550 | 0.9696 |
| GGT |  | 0.0014 | 0.8630 | 0.9308 | 0.0092 | 0.0820 | 0.5697 | 0.0094 | 0.3880 | 0.8369 |
| Glu0 |  | 0.0062 | 0.1100 | 0.5754 | 0.0122 | 0.0390 | 0.4538 | 0.0106 | 0.3170 | 0.7794 |
| Glu120 |  | 0.0320 | 0.0010 | 0.0340 | 0.0284 | 0.0020 | 0.0880 | 0.0215 | 0.0940 | 0.5238 |
| HbA1c |  | 0.0053 | 0.1410 | 0.6392 | 0.0046 | 0.3690 | 0.9014 | 0.0073 | 0.5750 | 0.9506 |
| Urine albumin |  | 0.0017 | 0.7470 | 0.9121 | 0.0023 | 0.7140 | 0.9762 | 0.0048 | 0.6820 | 0.9506 |
| *adjusted with Benjamini-Hochberg method. | | | | | | | | | | |

**Supplementary Table S3. Potential plasma biomarkers for discriminating Pre-diabetes group from T2D group.**

| **ID** | **mz** | **rt(min)** | **ppm** | **Ion** | **Database_ID** | **FORMULA** | **Name** | **Super_class** | **PATHWAY** | **Mean**  **(Pre-diabetes)** | **Mean(T2D)** | **Vip** | **Foldchange2**  **(T2D/Prediabetes)** | **pvalue** | **qvalue** |  |
| --- | --- | --- | --- | --- | --- | --- | --- | --- | --- | --- | --- | --- | --- | --- | --- | --- |
| NEG2879 | 282.08 | 2.29 | 0.07 | H- | C00387 | C10H13N5O5 | Guanosine(-) | Nucleosides, Nucleotides, and Analogues | ko00230 Purine metabolism | 1.88E+05 | 4.20E+05 | 1.56 | 2.24 | 2.44E-16 | 4.85E-16 |  |
| NEG4689 | 608.32 | 7.30 | 0.44 | CH3COO- | LMGP03050021 | C26H48NO9P | PS(20:2(11Z,14Z)/0:0)(-) | Lipids | Not_Available | 2.64E+05 | 1.52E+05 | 1.18 | 0.57 | 1.39E-13 | 2.21E-13 |  |
| POS3013 | 528.31 | 6.83 | 5.03 | H+ | HMDB11494 | C27H46NO7P | LysoPE(0:0/22:5(4Z,7Z,10Z,13Z,16Z))(+) | Lipids | Not_Available | 3.33E+05 | 5.17E+05 | 1.49 | 1.55 | 1.96E-14 | 3.30E-14 |  |
| NEG4342 | 526.29 | 7.00 | 0.25 | H- | HMDB11494 | C27H46NO7P | LysoPE(0:0/22:5(4Z,7Z,10Z,13Z,16Z))(-) | Lipids | Not_Available | 4.50E+05 | 7.60E+05 | 1.31 | 1.69 | 4.99E-16 | 9.64E-16 |  |
| NEG4242 | 502.29 | 6.91 | 0.12 | H- | HMDB11484 | C25H46NO7P | LysoPE(0:0/20:3(11Z,14Z,17Z))(-) | Lipids | Not_Available | 5.14E+05 | 9.30E+05 | 1.47 | 1.81 | 5.20E-19 | 1.37E-18 |  |
| NEG4388 | 536.32 | 6.35 | 8.65 | NAN | LMGP04050024 | C26H49O9P | PG(20:2(11Z,14Z)/0:0)(-) | Lipids | Not_Available | 5.72E+05 | 7.76E+05 | 1.00 | 1.36 | 7.29E-07 | 7.44E-07 |  |
| POS2157 | 303.18 | 7.56 | 2.79 | H+ | HMDB28707 | C11H22N6O4 | Glu Arg(+) | Amino Acids, Peptides, and Analogues | Not_Available | 5.97E+05 | 3.03E+05 | 1.49 | 0.51 | 8.73E-22 | 3.69E-21 |  |
| NEG4060 | 460.28 | 6.84 | 1.40 | (H2O+H)- | HMDB11475 | C23H46NO7P | LysoPE(0:0/18:1(11Z))(-) | Lipids | Not_Available | 9.43E+05 | 1.96E+05 | 2.54 | 0.21 | 1.35E-23 | 1.04E-22 |  |
| NEG2672 | 267.07 | 2.26 | 0.56 | H- | C00294 | C10H12N4O5 | Inosine(-) | Nucleosides, Nucleotides, and Analogues | ko00230 Purine metabolism | 9.55E+05 | 4.10E+06 | 2.36 | 4.29 | 6.44E-23 | 3.89E-22 |  |
| POS2891 | 500.27 | 6.67 | 8.42 | H+ | HMDB11489 | C25H42NO7P | LysoPE(0:0/20:5(5Z,8Z,11Z,14Z,17Z))(+) | Lipids | Not_Available | 1.08E+06 | 1.76E+06 | 2.09 | 1.63 | 1.15E-21 | 4.75E-21 |  |
| POS3003 | 526.29 | 7.65 | 2.71 | NAN | HMDB00739 | C24H40N5O8 | Isodesmosine(+) | Organic Acids and Derivatives | Not_Available | 1.19E+06 | 6.57E+05 | 1.33 | 0.55 | 2.56E-22 | 1.26E-21 |  |
| POS2870 | 494.36 | 7.54 | 3.59 | H+ | LMGP01070007 | C25H52NO6P | PC(P-17:0/0:0)(+) | Lipids | Not_Available | 1.21E+06 | 1.61E+05 | 3.03 | 0.13 | 7.98E-25 | 1.42E-23 |  |
| NEG4336 | 524.35 | 7.32 | 8.84 | NAN | LMGP04070001 | C26H53O8P | PG(P-20:0/0:0)(-) | Lipids | Not_Available | 1.34E+06 | 2.18E+05 | 2.70 | 0.16 | 1.72E-25 | 7.82E-24 |  |
| NEG4504 | 560.32 | 6.37 | 8.00 | NAN | LMGP04050017 | C28H49O9P | PG(22:4(7Z,10Z,13Z,16Z)/0:0)(-) | Lipids | Not_Available | 1.36E+06 | 2.56E+06 | 1.11 | 1.88 | 1.03E-09 | 1.24E-09 |  |
| POS3056 | 536.37 | 7.49 | 3.75 | H+ | LMGP01020070 | C27H54NO7P | PC(O-16:0/3:1(2E))(+) | Lipids | Not_Available | 1.39E+06 | 6.70E+05 | 1.86 | 0.48 | 5.60E-24 | 5.27E-23 |  |
| POS2214 | 311.12 | 2.57 | 3.60 | H+ | HMDB11741 | C14H18N2O6 | Gamma-Glutamyltyrosine(+) | Amino Acids, Peptides, and Analogues | Not_Available | 1.41E+06 | 6.00E+05 | 1.31 | 0.42 | 4.39E-13 | 6.77E-13 |  |
| NEG4834 | 877.55 | 7.77 | 0.61 | CH3COO- | LMGP06020078 | C43H79O12P | PI(O-16:0/18:3(9Z,12Z,15Z))(-) | Lipids | Not_Available | 1.44E+06 | 2.94E+05 | 2.90 | 0.20 | 1.02E-21 | 4.27E-21 |  |
| POS2404 | 342.15 | 2.68 | 3.36 | H+ | HMDB28896 | C17H19N5O3 | His Trp(+) | Amino Acids, Peptides, and Analogues | Not_Available | 1.45E+06 | 4.50E+06 | 3.20 | 3.10 | 1.14E-14 | 1.96E-14 |  |
| NEG657 | 149.10 | 5.95 | 0.88 | H- | C09840 | C10H14O | Carvacrol(-) | others | Not_Available | 1.54E+06 | 2.55E+06 | 1.33 | 1.66 | 8.09E-23 | 4.70E-22 |  |
| NEG3975 | 439.30 | 7.08 | 8.41 | NAN | LMGP02060002 | C21H46NO6P | PE(O-16:0/0:0)(-) | Lipids | Not_Available | 1.66E+06 | 4.01E+05 | 2.62 | 0.24 | 6.67E-23 | 4.01E-22 |  |
| POS3148 | 560.33 | 5.76 | 0.29 | Na+ | LMGP01010612 | C26H52NO8P | PC(16:0/2:0)(+) | Lipids | Not_Available | 1.68E+06 | 2.10E+05 | 2.50 | 0.13 | 5.96E-23 | 3.65E-22 |  |
| POS2678 | 428.37 | 7.59 | 3.26 | H+ | HMDB00848 | C25H49NO4 | Stearoylcarnitine(+) | Lipids | Not_Available | 1.73E+06 | 2.60E+06 | 1.23 | 1.50 | 3.12E-14 | 5.16E-14 |  |
| NEG4399 | 538.35 | 7.15 | 0.37 | H- | LMGP03060001 | C26H54NO8P | PS(O-20:0/0:0)(-) | Lipids | Not_Available | 1.76E+06 | 4.77E+05 | 2.15 | 0.27 | 7.83E-25 | 1.41E-23 |  |
| NEG4833 | 863.56 | 8.19 | 9.71 | H- | LMGP06010074 | C45H85O13P | PI(14:0/22:1(11Z))(-) | Lipids | Not_Available | 1.79E+06 | 7.21E+05 | 1.29 | 0.40 | 4.80E-15 | 8.57E-15 |  |
| NEG4540 | 566.36 | 7.32 | 7.92 | NAN | LMGP04050026 | C28H55O9P | PG(22:1(11Z)/0:0)(-) | Lipids | Not_Available | 1.98E+06 | 1.05E+06 | 1.48 | 0.53 | 2.76E-24 | 3.09E-23 |  |
| POS3001 | 525.36 | 7.15 | 7.91 | NH4+ | LMGP01050002 | C25H50NO7P | PC(17:1(10Z)/0:0)(+) | Lipids | Not_Available | 2.07E+06 | 1.46E+06 | 1.03 | 0.70 | 9.73E-20 | 2.87E-19 |  |
| POS1144 | 190.09 | 2.48 | 2.61 | H+ | HMDB02302 | C11H11NO2 | 3-Indolepropionic acid(+) | Aromatic Heteropolycyclic Compounds | Not_Available | 2.13E+06 | 9.39E+05 | 1.36 | 0.44 | 1.95E-19 | 5.50E-19 |  |
| POS2877 | 496.37 | 7.57 | 3.11 | H+ | LMGP01040030 | C25H54NO6P | PC(O-16:0/O-1:0)(+) | Lipids | Not_Available | 2.17E+06 | 2.34E+05 | 3.19 | 0.11 | 8.43E-25 | 1.46E-23 |  |
| NEG4841 | 903.56 | 7.86 | 4.04 | CH3COO- | LMGP06020032 | C45H81O12P | PI(O-18:0/18:4(6Z,9Z,12Z,15Z))(-) | Lipids | Not_Available | 2.18E+06 | 4.67E+05 | 2.15 | 0.21 | 1.21E-22 | 6.64E-22 |  |
| POS1405 | 219.13 | 1.45 | 2.42 | H+ | HMDB29042 | C9H18N2O4 | Ser Leu(+) | Amino Acids, Peptides, and Analogues | Not_Available | 2.38E+06 | 6.09E+05 | 2.23 | 0.26 | 8.90E-11 | 1.15E-10 |  |
| POS3198 | 572.37 | 7.23 | 3.70 | H+ | HMDB10401 | C30H54NO7P | LysoPC(22:4(7Z,10Z,13Z,16Z))(+) | Lipids | Not_Available | 2.47E+06 | 1.19E+06 | 1.56 | 0.48 | 3.31E-24 | 3.54E-23 |  |
| NEG4562 | 572.29 | 5.79 | 6.33 | NAN | LMGP06050002 | C25H49O12P | PI(16:0/0:0)(-) | Lipids | Not_Available | 2.51E+06 | 4.02E+05 | 2.82 | 0.16 | 1.76E-23 | 1.29E-22 |  |
| NEG4108 | 466.33 | 7.85 | 1.89 | H- | LMGP02060003 | C23H50NO6P | PE(O-18:0/0:0)(-) | Lipids | Not_Available | 2.77E+06 | 4.00E+05 | 2.71 | 0.14 | 2.26E-24 | 2.70E-23 |  |
| POS3059 | 536.41 | 8.22 | 3.85 | H+ | LMGP01040056 | C28H58NO6P | PC(O-18:0/O-2:1(1E))(+) | Lipids | Not_Available | 3.12E+06 | 3.00E+05 | 3.21 | 0.10 | 7.40E-26 | 6.53E-24 |  |
| POS3054 | 536.33 | 5.55 | 3.76 | H+ | LMGP01010693 | C26H50NO8P | PC(16:1(9Z)/2:0)(+) | Lipids | Not_Available | 3.19E+06 | 6.15E+05 | 2.48 | 0.19 | 3.40E-21 | 1.27E-20 |  |
| NEG4480 | 555.33 | 5.53 | 0.99 | CH3COO- | LMGP04070002 | C24H49O8P | PG(P-18:0/0:0)(-) | Lipids | Not_Available | 3.29E+06 | 2.26E+06 | 2.72 | 0.69 | 7.50E-19 | 1.93E-18 |  |
| POS2080 | 295.13 | 3.09 | 3.02 | H+ | HMDB00594 | C14H18N2O5 | Phe Glu(+) | Amino Acids, Peptides, and Analogues | Not_Available | 3.36E+06 | 1.43E+06 | 1.62 | 0.43 | 7.71E-16 | 1.47E-15 |  |
| POS1816 | 269.16 | 1.48 | 2.90 | H+ | C05010 | C12H20N4O3 | Leu His(+) | Amino Acids, Peptides, and Analogues | Not_Available | 3.39E+06 | 4.24E+05 | 2.61 | 0.12 | 5.52E-15 | 9.79E-15 |  |
| NEG4843 | 917.54 | 7.46 | 0.48 | CH3COO- | HMDB09789 | C45H79O13P | PI(16:0/20:4(5Z,8Z,11Z,14Z))(-) | Lipids | Not_Available | 3.45E+06 | 1.85E+06 | 2.20 | 0.54 | 1.11E-20 | 3.81E-20 |  |
| NEG4654 | 597.30 | 5.93 | 0.51 | H- | LMGP06050005 | C27H51O12P | PI(18:1(9Z)/0:0)(-) | Lipids | Not_Available | 3.55E+06 | 1.31E+06 | 1.77 | 0.37 | 3.69E-22 | 1.72E-21 |  |
| NEG4825 | 835.53 | 8.14 | 0.79 | H- | HMDB09782 | C43H81O13P | PI(16:0/18:1(11Z))(-) | Lipids | Not_Available | 4.00E+06 | 2.07E+06 | 1.31 | 0.52 | 2.22E-17 | 4.83E-17 |  |
| POS2260 | 316.25 | 5.29 | 3.13 | H+ | HMDB00651 | C17H33NO4 | Decanoylcarnitine(+) | Lipids | Not_Available | 4.26E+06 | 9.66E+06 | 1.79 | 2.27 | 2.94E-20 | 9.38E-20 |  |
| POS1871 | 276.12 | 0.58 | 2.72 | H+ | HMDB11738 | C10H17N3O6 | Glu Gln(+) | Amino Acids, Peptides, and Analogues | Not_Available | 4.43E+06 | 7.09E+06 | 1.33 | 1.60 | 1.20E-10 | 1.53E-10 |  |
| POS2124 | 300.29 | 6.50 | 3.01 | H+ | C00319 | C18H37NO2 | Sphingosine(+) | Lipids | ko00600 Sphingolipid metabolism | 4.47E+06 | 2.42E+06 | 1.34 | 0.54 | 2.62E-16 | 5.20E-16 |  |
| NEG4823 | 833.52 | 8.01 | 0.62 | H- | HMDB09784 | C43H79O13P | PI(16:0/18:2(9Z,12Z))(-) | Lipids | Not_Available | 4.58E+06 | 2.79E+06 | 1.08 | 0.61 | 2.19E-13 | 3.44E-13 |  |
| POS3004 | 526.29 | 6.66 | 3.45 | H+ | HMDB11496 | C27H44NO7P | LysoPE(0:0/22:6(4Z,7Z,10Z,13Z,16Z,19Z))(+) | Lipids | Not_Available | 4.69E+06 | 8.77E+06 | 1.66 | 1.87 | 1.52E-22 | 8.01E-22 |  |
| NEG2559 | 259.13 | 0.89 | 0.37 | H- | HMDB11170 | C11H20N2O5 | Gamma-Glu-Leu(-) | Amino Acids, Peptides, and Analogues | Not_Available | 4.74E+06 | 1.58E+06 | 2.24 | 0.33 | 6.39E-09 | 7.35E-09 |  |
| NEG4333 | 524.28 | 6.66 | 0.04 | H- | HMDB11496 | C27H44NO7P | LysoPE(0:0/22:6(4Z,7Z,10Z,13Z,16Z,19Z))(-) | Lipids | Not_Available | 4.99E+06 | 9.31E+06 | 1.66 | 1.87 | 4.02E-22 | 1.86E-21 |  |
| POS1872 | 276.15 | 0.55 | 2.85 | H+ | HMDB03869 | C11H21N3O5 | Glu Lys(+) | Amino Acids, Peptides, and Analogues | Not_Available | 5.17E+06 | 1.99E+06 | 1.48 | 0.38 | 6.26E-09 | 7.20E-09 |  |
| POS1881 | 277.10 | 0.60 | 2.82 | H+ | HMDB11737 | C10H16N2O7 | Glu Glu(+) | Amino Acids, Peptides, and Analogues | Not_Available | 5.35E+06 | 1.89E+06 | 1.72 | 0.35 | 1.02E-14 | 1.77E-14 |  |
| POS2936 | 511.39 | 8.05 | 5.22 | K+ | LMFA01030827 | C32H56O2 | LysoPC(32:4(17Z,20Z,23Z,26Z))(+) | Lipids | Not_Available | 5.76E+06 | 9.51E+05 | 2.60 | 0.17 | 1.12E-25 | 7.07E-24 |  |
| POS2897 | 502.29 | 6.68 | 3.64 | H+ | HMDB11487 | C25H44NO7P | LysoPE(0:0/20:4(5Z,8Z,11Z,14Z))(+) | Lipids | Not_Available | 5.77E+06 | 8.66E+06 | 1.30 | 1.50 | 2.73E-18 | 6.57E-18 |  |
| POS2814 | 478.29 | 6.66 | 3.42 | H+ | HMDB11477 | C23H44NO7P | LysoPE(0:0/18:2(9Z,12Z))(+) | Lipids | Not_Available | 6.19E+06 | 1.32E+07 | 1.87 | 2.14 | 6.44E-23 | 3.89E-22 |  |
| POS326 | 118.07 | 2.48 | 0.42 | H+ | C00463 | C8H7N | Indole(+) | Aromatic Heteropolycyclic Compounds | ko00380 Tryptophan metabolism | 7.93E+06 | 4.75E+06 | 1.09 | 0.60 | 1.52E-17 | 3.37E-17 |  |
| NEG4839 | 901.55 | 7.84 | 0.54 | CH3COO- | LMGP06020075 | C45H79O12P | PI(O-16:0/20:5(5Z,8Z,11Z,14Z,17Z))(-) | Lipids | Not_Available | 8.65E+06 | 2.04E+06 | 3.47 | 0.24 | 1.70E-23 | 1.25E-22 |  |
| POS3109 | 548.37 | 7.34 | 3.63 | H+ | HMDB10392 | C28H54NO7P | LysoPC(20:2(11Z,14Z))(+) | Lipids | Not_Available | 9.12E+06 | 4.43E+06 | 1.62 | 0.49 | 3.41E-25 | 9.24E-24 |  |
| NEG4140 | 476.28 | 6.66 | 0.02 | H- | HMDB11477 | C23H44NO7P | LysoPE(0:0/18:2(9Z,12Z))(-) | Lipids | Not_Available | 9.18E+06 | 1.70E+07 | 1.83 | 1.85 | 6.98E-22 | 3.04E-21 |  |
| POS3113 | 550.38 | 7.87 | 3.44 | H+ | HMDB10391 | C28H56NO7P | LysoPC(20:1(11Z))(+) | Lipids | Not_Available | 9.55E+06 | 4.13E+06 | 1.79 | 0.43 | 1.12E-25 | 7.07E-24 |  |
| POS2922 | 508.37 | 7.96 | 3.12 | H+ | HMDB13122 | C26H54NO6P | LysoPC(P-18:0)(+) | Lipids | Not_Available | 9.80E+06 | 2.01E+06 | 2.40 | 0.20 | 1.40E-25 | 7.45E-24 |  |
| NEG4221 | 498.34 | 7.15 | 9.33 | NAN | LMGP04060002 | C24H51O8P | PG(O-18:0/0:0)(-) | Lipids | Not_Available | 1.04E+07 | 2.96E+06 | 2.10 | 0.29 | 3.83E-24 | 3.94E-23 |  |
| NEG4092 | 465.30 | 7.40 | 0.20 | H- | C18043 | C27H46O4S | Cholesterol sulfate(-) | Lipids | ko00140 Steroid hormone biosynthesis | 1.07E+07 | 6.99E+06 | 1.01 | 0.65 | 3.14E-15 | 5.72E-15 |  |
| POS212 | 106.05 | 0.50 | 1.45 | H+ | C00065 | C3H7NO3 | L-Serine(+)* | Amino Acids, Peptides, and Analogues | ko00260 Glycine, serine and threonine metabolism | 1.13E+07 | 5.54E+06 | 1.38 | 0.49 | 5.86E-20 | 1.79E-19 |  |
| POS512 | 134.04 | 0.51 | 0.97 | H+ | C00049 | C4H7NO4 | L-Aspartate(+)* | Amino Acids, Peptides, and Analogues | ko00250 Alanine, aspartate and glutamate metabolism | 1.18E+07 | 3.90E+06 | 1.69 | 0.33 | 2.27E-21 | 8.81E-21 |  |
| POS1750 | 261.14 | 2.94 | 2.46 | H+ | HMDB11170 | C11H20N2O5 | Gamma-Glu-Leu(+) | Amino Acids, Peptides, and Analogues | Not_Available | 1.38E+07 | 5.21E+06 | 1.75 | 0.38 | 1.01E-14 | 1.74E-14 |  |
| NEG3098 | 301.22 | 6.97 | 0.03 | H- | HMDB01999 | C20H30O2 | Eicosapentaenoic acid(-) | Lipids | Not_Available | 1.40E+07 | 1.69E+06 | 3.27 | 0.12 | 1.02E-22 | 5.70E-22 |  |
| POS2237 | 313.15 | 3.72 | 3.30 | H+ | HMDB13302 | C18H20N2O3 | Phe Phe(+) | Amino Acids, Peptides, and Analogues | Not_Available | 1.75E+07 | 5.30E+07 | 2.80 | 3.03 | 3.30E-21 | 1.24E-20 |  |
| POS2932 | 510.39 | 8.05 | 3.11 | H+ | HMDB11149 | C26H56NO6P | LysoPC(O-18:0)(+) | Lipids | Not_Available | 2.02E+07 | 3.63E+06 | 2.52 | 0.18 | 1.51E-25 | 7.59E-24 |  |
| POS2930 | 510.35 | 7.32 | 3.32 | H+ | HMDB12108 | C25H52NO7P | LysoPC(17:0)(+) | Lipids | Not_Available | 2.72E+07 | 1.49E+07 | 1.42 | 0.55 | 5.21E-23 | 3.26E-22 |  |
| NEG3268 | 317.21 | 7.04 | 0.05 | H- | HMDB05081 | C20H30O3 | 5-HEPE(-) | Lipids | Not_Available | 3.20E+07 | 3.54E+06 | 2.96 | 0.11 | 2.66E-23 | 1.82E-22 |  |
| NEG1081 | 172.08 | 1.05 | 3.71 | (H2O+H)- | HMDB01896 | C11H13NO2 | 5-Methoxytryptophol(-) | Aromatic Heteropolycyclic Compounds | Not_Available | 3.25E+07 | 1.94E+07 | 1.24 | 0.60 | 2.86E-18 | 6.86E-18 |  |
| POS884 | 166.05 | 0.53 | 1.85 | H+ | C02989 | C5H11NO3S | L-Methionine S-oxide(+) | Amino Acids, Peptides, and Analogues | ko00270 Cysteine and methionine metabolism | 3.52E+07 | 2.28E+06 | 2.81 | 0.06 | 1.19E-22 | 6.53E-22 |  |
| POS2828 | 482.32 | 7.65 | 3.27 | H+ | HMDB11129 | C23H48NO7P | LysoPE(0:0/18:0)(+) | Lipids | Not_Available | 3.56E+07 | 1.73E+07 | 1.49 | 0.48 | 1.23E-23 | 9.77E-23 |  |
| POS660 | 147.08 | 0.51 | 1.45 | H+ | C00064 | C5H10N2O3 | L-Glutamine(+)* | Amino Acids, Peptides, and Analogues | ko00230 Purine metabolism | 3.60E+07 | 7.22E+07 | 1.69 | 2.01 | 6.40E-19 | 1.66E-18 |  |
| POS347 | 120.07 | 0.52 | 0.39 | H+ | C00263 | C4H9NO3 | L-Homoserine(+) | Amino Acids, Peptides, and Analogues | ko00260 Glycine, serine and threonine metabolism | 4.19E+07 | 2.27E+07 | 1.14 | 0.54 | 1.82E-17 | 4.00E-17 |  |
| NEG3952 | 436.28 | 7.11 | 0.13 | H- | HMDB11152 | C21H44NO6P | PE(P-16:0e/0:0)(-) | Lipids | Not_Available | 4.29E+07 | 9.31E+06 | 2.36 | 0.22 | 3.37E-24 | 3.58E-23 |  |
| NEG4079 | 464.31 | 7.84 | 0.14 | H- | LMGP02060004 | C23H48NO6P | PE(O-18:1(9Z)/0:0)(-) | Lipids | Not_Available | 4.53E+07 | 9.82E+06 | 2.36 | 0.22 | 2.97E-24 | 3.27E-23 |  |
| NEG3030 | 295.23 | 6.50 | 0.21 | H- | C14762 | C18H32O3 | 13(S)-HODE(-) | Lipids | Not_Available | 4.56E+07 | 6.33E+06 | 2.47 | 0.14 | 6.32E-23 | 3.84E-22 |  |
| NEG4158 | 480.31 | 7.61 | 0.02 | H- | C23H48NO7P | HMDB11130 | LysoPE(18:0/0:0)(-) | Lipids | Not_Available | 4.75E+07 | 2.54E+07 | 1.32 | 0.54 | 1.94E-20 | 6.41E-20 |  |
| NEG620 | 145.06 | 0.52 | 0.47 | H- | C00064 | C5H10N2O3 | L-Glutamine(-)* | Amino Acids, Peptides, and Analogues | ko00471 D-Glutamine and D-glutamate metabolism | 4.91E+07 | 9.61E+07 | 1.60 | 1.96 | 1.71E-18 | 4.23E-18 |  |
| NEG2738 | 271.23 | 6.56 | 0.02 | H- | HMDB31057 | C16H32O3 | 2-hydroxyhexadecanoic acid(-) | Lipids | Not_Available | 5.08E+07 | 2.90E+07 | 1.24 | 0.57 | 3.77E-15 | 6.82E-15 |  |
| POS2819 | 480.34 | 7.17 | 3.26 | H+ | HMDB10407 | C24H50NO6P | LysoPC(P-16:0)(+) | Lipids | Not_Available | 6.29E+07 | 1.50E+07 | 2.31 | 0.24 | 7.40E-26 | 6.53E-24 |  |
| NEG4661 | 599.32 | 6.26 | 0.53 | H- | LMGP06050004 | C27H53O12P | PI(18:0/0:0)(-) | Lipids | Not_Available | 6.39E+07 | 1.05E+07 | 2.57 | 0.16 | 1.48E-21 | 5.99E-21 |  |
| NEG3434 | 335.22 | 5.66 | 0.20 | H- | C00959 | C20H32O4 | Prostaglandin B1(-) | Lipids | Not_Available | 6.81E+07 | 8.55E+06 | 3.18 | 0.13 | 1.26E-23 | 9.89E-23 |  |
| NEG3850 | 409.24 | 6.07 | 0.02 | H- | HMDB07849 | C19H39O7P | 1-Palmitoyl Lysophosphatidic Acid(-) | Lipids | Not_Available | 8.49E+07 | 1.24E+07 | 2.57 | 0.15 | 7.14E-25 | 1.34E-23 |  |
| POS2829 | 482.36 | 7.16 | 2.88 | H+ | LMGP01060040 | C24H52NO6P | PC(O-16:0/0:0)(+) | Lipids | Not_Available | 8.54E+07 | 1.73E+07 | 2.45 | 0.20 | 2.45E-25 | 8.37E-24 |  |
| NEG1435 | 191.02 | 0.44 | 0.12 | H- | C00158 | C6H8O7 | Citrate(-) | Organic Acids and Derivatives | ko00020 Citrate cycle (TCA cycle) | 8.66E+07 | 2.34E+07 | 1.72 | 0.27 | 4.29E-18 | 1.01E-17 |  |
| POS661 | 147.11 | 0.45 | 1.51 | H+ | C00047 | C6H14N2O2 | L-Lysine(+)* | Amino Acids, Peptides, and Analogues | ko00300 Lysine biosynthesis | 1.13E+08 | 4.76E+07 | 1.26 | 0.42 | 1.50E-17 | 3.32E-17 |  |
| POS453 | 130.05 | 1.05 | 0.83 | H+ | C01879 | C5H7NO3 | Pyroglutamic acid(+) | Aliphatic Heteromonocyclic Compounds | ko00480 Glutathione metabolism | 1.24E+08 | 2.99E+07 | 2.25 | 0.24 | 1.89E-24 | 2.39E-23 |  |
| NEG718 | 154.06 | 0.58 | 0.27 | H- | C00135 | C6H9N3O2 | L-Histidine(-)* | Amino Acids, Peptides, and Analogues | ko00340 Histidine metabolism | 1.39E+08 | 5.67E+07 | 1.62 | 0.41 | 1.42E-22 | 7.56E-22 |  |
| NEG447 | 132.03 | 0.45 | 0.10 | H- | C00049 | C4H7NO4 | L-Aspartate(-)* | Amino Acids, Peptides, and Analogues | ko00250 Alanine, aspartate and glutamate metabolism | 1.73E+08 | 4.78E+07 | 1.83 | 0.28 | 2.22E-20 | 7.25E-20 |  |
| POS2999 | 524.37 | 7.56 | 2.89 | H+ | LMGP01020046 | C26H54NO7P | PC(O-16:0/2:0)(+) | Lipids | Not_Available | 1.93E+08 | 1.01E+08 | 1.47 | 0.53 | 4.54E-21 | 1.66E-20 |  |
| POS668 | 148.06 | 0.53 | 2.13 | H+ | C00025 | C5H9NO4 | L-Glutamate(+)* | Amino Acids, Peptides, and Analogues | ko00250 Alanine, aspartate and glutamate metabolism | 1.96E+08 | 6.98E+07 | 1.82 | 0.36 | 6.98E-22 | 3.04E-21 |  |
| NEG1243 | 180.07 | 1.01 | 0.06 | H- | C00082 | C9H11NO3 | L-Tyrosine(-)* | Amino Acids, Peptides, and Analogues | ko00130 Ubiquinone and other terpenoid-quinone biosynthesis | 2.85E+08 | 1.37E+08 | 1.17 | 0.48 | 2.17E-15 | 4.00E-15 |  |
| POS2982 | 522.35 | 7.14 | 3.24 | H+ | HMDB02815 | C26H52NO7P | LysoPC(18:1(9Z))(+) | Lipids | Not_Available | 3.13E+08 | 2.16E+08 | 1.02 | 0.69 | 9.73E-20 | 2.87E-19 |  |
| NEG3499 | 343.23 | 6.88 | 0.20 | H- | HMDB60050 | C22H32O3 | 7-HDoHE(-) | Lipids | Not_Available | 3.64E+08 | 2.97E+07 | 3.06 | 0.08 | 6.41E-22 | 2.82E-21 |  |
| POS1062 | 182.08 | 1.58 | 1.85 | H+ | C00082 | C9H11NO3 | L-Tyrosine(+)* | Amino Acids, Peptides, and Analogues | ko00400 Phenylalanine, tyrosine and tryptophan biosynthesis | 3.70E+08 | 1.85E+08 | 1.10 | 0.50 | 3.29E-14 | 5.44E-14 |  |
| POS839 | 162.11 | 0.54 | 1.95 | H+ | C00318 | C7H15NO3 | L-Carnitine(+) | Amino Acids, Peptides, and Analogues | ko04976 Bile secretion | 3.80E+08 | 5.21E+08 | 1.10 | 1.37 | 6.91E-23 | 4.13E-22 |  |
| NEG895 | 164.07 | 2.33 | 0.08 | H- | C00079 | C9H11NO2 | L-Phenylalanine(-)* | Amino Acids, Peptides, and Analogues | ko00400 Phenylalanine, tyrosine and tryptophan biosynthesis | 4.91E+08 | 2.36E+08 | 1.31 | 0.48 | 4.36E-19 | 1.17E-18 |  |
| NEG625 | 146.05 | 0.45 | 0.03 | H- | C00025 | C5H9NO4 | L-Glutamate(-)* | Amino Acids, Peptides, and Analogues | ko00471 D-Glutamine and D-glutamate metabolism | 5.17E+08 | 1.93E+08 | 1.78 | 0.37 | 2.01E-20 | 6.61E-20 |  |
| POS886 | 166.09 | 2.48 | 1.93 | H+ | C00079 | C9H11NO2 | L-Phenylalanine(+)* | Amino Acids, Peptides, and Analogues | ko00400 Phenylalanine, tyrosine and tryptophan biosynthesis | 1.05E+09 | 4.99E+08 | 1.31 | 0.48 | 2.26E-19 | 6.29E-19 |  |
| POS2876 | 496.34 | 6.94 | 3.07 | H+ | HMDB10382 | C24H50NO7P | LysoPC(16:0)(+) | Lipids | Not_Available | 1.90E+09 | 1.31E+09 | 1.09 | 0.69 | 6.30E-22 | 2.78E-21 |  |
| *Metabolites matched with commercial available reference standards; other metabolites were matched with the online database. | | | | | | | | | | | | | | | | |

**Supplementary Table S4. The MetPA was applied for significant metabolites distinguishing Pre-diabetes group from T2D group.**

| **Pathway_name** | **Pre-diabetes group VS T2D group** | | | |
| --- | --- | --- | --- | --- |
|  | **Raw p*** | **Holm p**† | **FDR p**‡ | **Impact**§ |
| Aminoacyl-tRNA biosynthesis | 0.0000 | 0.0000 | 0.0000 | 0.1667 |
| Alanine, aspartate and glutamate metabolism | 0.0008 | 0.0690 | 0.0247 | 0.5345 |
| Arginine biosynthesis | 0.0012 | 0.0985 | 0.0247 | 0.1168 |
| Glyoxylate and dicarboxylate metabolism | 0.0014 | 0.1132 | 0.0247 | 0.0741 |
| Phenylalanine, tyrosine and tryptophan biosynthesis | 0.0015 | 0.1176 | 0.0247 | 1.0000 |
| Histidine metabolism | 0.0018 | 0.1429 | 0.0253 | 0.2213 |
| Nitrogen metabolism | 0.0036 | 0.2810 | 0.0378 | 0.0000 |
| D-Glutamine and D-glutamate metabolism | 0.0036 | 0.2810 | 0.0378 | 0.5000 |
| Phenylalanine metabolism | 0.0104 | 0.7894 | 0.0969 | 0.3571 |
| beta-Alanine metabolism | 0.0435 | 1.0000 | 0.3321 | 0.0000 |
| Sphingolipid metabolism | 0.0435 | 1.0000 | 0.3321 | 0.0446 |
| Glutathione metabolism | 0.0731 | 1.0000 | 0.5118 | 0.0268 |
| Purine metabolism | 0.0839 | 1.0000 | 0.5420 | 0.0023 |
| Ubiquinone and other terpenoid-quinone biosynthesis | 0.1365 | 1.0000 | 0.8188 | 0.0000 |
| Biotin metabolism | 0.1505 | 1.0000 | 0.8427 | 0.0000 |
| Butanoate metabolism | 0.2173 | 1.0000 | 1.0000 | 0.0000 |
| Nicotinate and nicotinamide metabolism | 0.2173 | 1.0000 | 1.0000 | 0.0000 |
| Pantothenate and CoA biosynthesis | 0.2671 | 1.0000 | 1.0000 | 0.0000 |
| Citrate cycle (TCA cycle) | 0.2791 | 1.0000 | 1.0000 | 0.0904 |
| Ether lipid metabolism | 0.2791 | 1.0000 | 1.0000 | 0.1446 |
| Lysine degradation | 0.3362 | 1.0000 | 1.0000 | 0.0000 |
| Porphyrin and chlorophyll metabolism | 0.3889 | 1.0000 | 1.0000 | 0.0000 |
| Cysteine and methionine metabolism | 0.4186 | 1.0000 | 1.0000 | 0.0218 |
| Glycine, serine and threonine metabolism | 0.4186 | 1.0000 | 1.0000 | 0.2171 |
| Biosynthesis of unsaturated fatty acids | 0.4469 | 1.0000 | 1.0000 | 0.0000 |
| Glycerophospholipid metabolism | 0.4469 | 1.0000 | 1.0000 | 0.0174 |
| Arginine and proline metabolism | 0.4650 | 1.0000 | 1.0000 | 0.0860 |
| Pyrimidine metabolism | 0.4738 | 1.0000 | 1.0000 | 0.0000 |
| Tyrosine metabolism | 0.4995 | 1.0000 | 1.0000 | 0.1397 |
| Steroid hormone biosynthesis | 0.7586 | 1.0000 | 1.0000 | 0.0000 |
| *The Raw p is the original p value calculated from the enrichment analysis;  †the Holm p is the p value adjusted by Holm-Bonferroni method;  ‡the FDR p is the p value adjusted using False Discovery Rate;  §the Impact is the pathway impact value calculated from pathway topology analysis. | | | | |
|  |  |  |  |  |
|  |  |  |  |  |
|  |  |  |  |  |

**Supplementary Table S5. A multi-logistic regression analysis to estimate the OR per SD increment and 95% CI for**

**the association between each novel metabolite and T2D risk.**

| **Variables*** | | **Pre-diabetes group VS T2D group** | | | | |
| --- | --- | --- | --- | --- | --- | --- |
|  |  | **OR** | **0.025** | **0.975** | **P-value** | **Q-value** |
| HbA1c | A1C | 0.4353 | 0.1173 | 1.6155 | 0.2138 | 0.2287 |
| ALT | ALT | 1.0245 | 0.9651 | 1.0875 | 0.4278 | 0.4349 |
| AST | AST | 0.9879 | 0.9154 | 1.0662 | 0.7546 | 0.7608 |
| BMI | BMI | 0.8203 | 0.6023 | 1.1172 | 0.2088 | 0.2253 |
| TC | CHOL | 0.6832 | 0.3250 | 1.4361 | 0.3149 | 0.3228 |
| DBP | DBP | 0.9672 | 0.9083 | 1.0298 | 0.2971 | 0.3112 |
| GGT | GGT | 0.9845 | 0.9672 | 1.0021 | 0.0831 | 0.0921 |
| Glu0 | Glu0 | 6.2780 | 2.8842 | 13.6649 | 0.0000 | 0.0000 |
| Glu120 | Glu120 | 5.6410 | 3.5479 | 8.9690 | 0.0000 | 0.0000 |
| HC | HC | 1.4105 | 0.7892 | 2.5210 | 0.2457 | 0.2605 |
| HDL-C | HDL | 3.7790 | 0.6344 | 22.5108 | 0.1442 | 0.1570 |
| HR | HR | 0.9745 | 0.9283 | 1.0231 | 0.2986 | 0.3112 |
| LDL-C | LDL | 2.4707 | 0.9829 | 6.2102 | 0.0544 | 0.0620 |
| SBP | SBP | 0.9488 | 0.9072 | 0.9923 | 0.0215 | 0.0252 |
| TG | TG | 0.3951 | 0.2039 | 0.7659 | 0.0060 | 0.0071 |
| Urine.albumin | Urine.ALB | 1.0468 | 0.9987 | 1.0972 | 0.0567 | 0.0640 |
| Waist.hip.ratio | Waist.hip.ratio | 1.1780 | 0.0000 | 68564.8063 | 0.9766 | 0.9766 |
| WC | WC | 1.1189 | 0.9870 | 1.2685 | 0.0792 | 0.0885 |
| Age | Age | 1.1009 | 1.0316 | 1.1749 | 0.0038 | 0.0045 |
| Gender | Gender | 1.7871 | 0.5832 | 5.4760 | 0.3095 | 0.3199 |
| NEG1081 | 5-Methoxytryptophol(-) | 0.0126 | 0.0022 | 0.0717 | 0.0000 | 0.0000 |
| NEG1243 | L-Tyrosine(-) | 0.0017 | 0.0001 | 0.0285 | 0.0000 | 0.0000 |
| NEG1435 | Citrate(-) | 0.0131 | 0.0021 | 0.0832 | 0.0000 | 0.0000 |
| NEG2559 | Gamma-Glu-Leu(-) | 0.0032 | 0.0001 | 0.0791 | 0.0005 | 0.0006 |
| NEG2672 | Inosine (-) | 19.0045 | 4.2306 | 85.3714 | 0.0001 | 0.0002 |
| NEG2738 | 2-hydroxyhexadecanoic acid(-) | 0.2239 | 0.1033 | 0.4852 | 0.0001 | 0.0002 |
| NEG2879 | Guanosine (-) | 4.2111 | 1.9475 | 9.1057 | 0.0003 | 0.0004 |
| NEG3030 | 13(S)-HODE(-) | 0.0037 | 0.0002 | 0.0739 | 0.0003 | 0.0004 |
| NEG3098 | Eicosapentaenoic acid(-) | 0.0168 | 0.0029 | 0.0972 | 0.0000 | 0.0000 |
| NEG3268 | 5-HEPE(-) | 0.0006 | 0.0000 | 0.0294 | 0.0002 | 0.0003 |
| NEG3434 | Prostaglandin B1(-) | 0.0094 | 0.0015 | 0.0581 | 0.0000 | 0.0000 |
| NEG3499 | 7-HDoHE(-) | 0.0066 | 0.0005 | 0.0832 | 0.0001 | 0.0002 |
| NEG3850 | 1-Palmitoyl Lysophosphatidic Acid(-) | 0.0002 | 0.0000 | 0.0104 | 0.0000 | 0.0000 |
| NEG3952 | PE(P-16:0e/0:0)(-) | 0.0049 | 0.0004 | 0.0537 | 0.0000 | 0.0000 |
| NEG3975 | PE(O-16:0/0:0)(-) | 0.0033 | 0.0002 | 0.0468 | 0.0000 | 0.0000 |
| NEG4060 | LysoPE(0:0/18:1(11Z))(-) | 0.0050 | 0.0004 | 0.0600 | 0.0000 | 0.0001 |
| NEG4079 | PE(O-18:1(9Z)/0:0)(-) | 0.0124 | 0.0020 | 0.0776 | 0.0000 | 0.0000 |
| NEG4092 | Cholesterol sulfate(-) | 0.2982 | 0.1645 | 0.5405 | 0.0001 | 0.0001 |
| NEG4108 | PE(O-18:0/0:0)(-) | 0.0034 | 0.0003 | 0.0406 | 0.0000 | 0.0000 |
| NEG4140 | LysoPE(0:0/18:2(9Z,12Z))(-) | 6.5231 | 2.9860 | 14.2501 | 0.0000 | 0.0000 |
| NEG4158 | LysoPE(18:0/0:0)(-) | 0.0682 | 0.0210 | 0.2219 | 0.0000 | 0.0000 |
| NEG4221 | PG(O-18:0/0:0)(-) | 0.0034 | 0.0002 | 0.0513 | 0.0000 | 0.0001 |
| NEG4242 | LysoPE(0:0/20:3(11Z,14Z,17Z))(-) | 3.9704 | 1.8966 | 8.3117 | 0.0003 | 0.0004 |
| NEG4333 | LysoPE(0:0/22:6(4Z,7Z,10Z,13Z,16Z,19Z))(-) | 9.4031 | 3.6849 | 23.9950 | 0.0000 | 0.0000 |
| NEG4336 | PG(P-20:0/0:0)(-) | 0.0018 | 0.0001 | 0.0454 | 0.0001 | 0.0002 |
| NEG4342 | LysoPE(0:0/22:5(4Z,7Z,10Z,13Z,16Z))(-) | 3.7870 | 1.9388 | 7.3969 | 0.0001 | 0.0002 |
| NEG4388 | PG(20:2(11Z,14Z)/0:0)(-) | 1.5387 | 1.0045 | 2.3571 | 0.0477 | 0.0548 |
| NEG4399 | PS(O-20:0/0:0)(-) | 0.0026 | 0.0001 | 0.0466 | 0.0001 | 0.0001 |
| NEG447 | L-Aspartate(-) | 0.0004 | 0.0000 | 0.0124 | 0.0000 | 0.0000 |
| NEG4480 | PG(P-18:0/0:0)(-) | 0.1125 | 0.0415 | 0.3050 | 0.0000 | 0.0000 |
| NEG4504 | PG(22:4(7Z,10Z,13Z,16Z)/0:0)(-) | 1.7965 | 1.0242 | 3.1509 | 0.0410 | 0.0476 |
| NEG4540 | PG(22:1(11Z)/0:0)(-) | 0.0537 | 0.0181 | 0.1592 | 0.0000 | 0.0000 |
| NEG4562 | PI(16:0/0:0)(-) | 0.0104 | 0.0014 | 0.0782 | 0.0000 | 0.0000 |
| NEG4654 | PI(18:1(9Z)/0:0)(-) | 0.0268 | 0.0060 | 0.1202 | 0.0000 | 0.0000 |
| NEG4661 | PI(18:0/0:0)(-) | 0.0060 | 0.0008 | 0.0420 | 0.0000 | 0.0000 |
| NEG4689 | PS(20:2(11Z,14Z)/0:0)(-) | 0.2091 | 0.1006 | 0.4349 | 0.0000 | 0.0001 |
| NEG4823 | PI(16:0/18:2(9Z,12Z))(-) | 0.2986 | 0.1603 | 0.5563 | 0.0001 | 0.0002 |
| NEG4825 | PI(16:0/18:1(11Z))(-) | 0.2010 | 0.1003 | 0.4031 | 0.0000 | 0.0000 |
| NEG4833 | PI(14:0/22:1(11Z))(-) | 0.0303 | 0.0043 | 0.2125 | 0.0004 | 0.0006 |
| NEG4834 | PI(O-16:0/18:3(9Z,12Z,15Z))(-) | 0.0541 | 0.0174 | 0.1677 | 0.0000 | 0.0000 |
| NEG4839 | PI(O-16:0/20:5(5Z,8Z,11Z,14Z,17Z))(-) | 0.0449 | 0.0137 | 0.1476 | 0.0000 | 0.0000 |
| NEG4841 | PI(O-18:0/18:4(6Z,9Z,12Z,15Z))(-) | 0.0611 | 0.0198 | 0.1888 | 0.0000 | 0.0000 |
| NEG4843 | PI(16:0/20:4(5Z,8Z,11Z,14Z))(-) | 0.0136 | 0.0020 | 0.0914 | 0.0000 | 0.0000 |
| NEG620 | L-Glutamine(-) | 4.2094 | 2.3094 | 7.6728 | 0.0000 | 0.0000 |
| NEG625 | L-Glutamate(-) | 0.0285 | 0.0076 | 0.1070 | 0.0000 | 0.0000 |
| NEG657 | Carvacrol (-) | 17.6278 | 4.9844 | 62.3430 | 0.0000 | 0.0000 |
| NEG718 | L-Histidine(-) | 0.0001 | 0.0000 | 0.0041 | 0.0000 | 0.0000 |
| NEG895 | L-Phenylalanine(-) | 0.0008 | 0.0000 | 0.0135 | 0.0000 | 0.0000 |
| POS1062 | L-Tyrosine(+) | 0.0029 | 0.0002 | 0.0448 | 0.0000 | 0.0001 |
| POS1144 | 3-Indolepropionic acid(+) | 0.0009 | 0.0001 | 0.0146 | 0.0000 | 0.0000 |
| POS1405 | Ser Leu(+) | 0.0404 | 0.0063 | 0.2584 | 0.0007 | 0.0009 |
| POS1503 | Ile Val(+) | 0.3822 | 0.1738 | 0.8402 | 0.0167 | 0.0198 |
| POS1750 | Gamma-Glu-Leu(+) | 0.1090 | 0.0374 | 0.3173 | 0.0000 | 0.0001 |
| POS1816 | Leu His(+) | 0.0016 | 0.0001 | 0.0316 | 0.0000 | 0.0000 |
| POS1871 | Glu Gln(+) | 2.1029 | 1.3500 | 3.2757 | 0.0010 | 0.0013 |
| POS1872 | Glu Lys(+) | 0.1067 | 0.0245 | 0.4643 | 0.0029 | 0.0035 |
| POS1881 | Glu Glu(+) | 0.1332 | 0.0437 | 0.4062 | 0.0004 | 0.0006 |
| POS1953 | Oleamide(+) | 0.2504 | 0.0394 | 1.5931 | 0.1425 | 0.1564 |
| POS2080 | Phe Glu(+) | 0.1759 | 0.0799 | 0.3868 | 0.0000 | 0.0000 |
| POS212 | L-Serine(+) | 0.0005 | 0.0000 | 0.0119 | 0.0000 | 0.0000 |
| POS2124 | Sphingosine(+) | 0.2407 | 0.1228 | 0.4715 | 0.0000 | 0.0001 |
| POS2157 | Glu Arg(+) | 0.0247 | 0.0054 | 0.1134 | 0.0000 | 0.0000 |
| POS2214 | Gamma-Glutamyltyrosine(+) | 0.0311 | 0.0046 | 0.2087 | 0.0004 | 0.0005 |
| POS2237 | Phe Phe(+) | 6.3632 | 3.2320 | 12.5280 | 0.0000 | 0.0000 |
| POS2260 | Decanoylcarnitine(+) | 15.2643 | 5.2847 | 44.0894 | 0.0000 | 0.0000 |
| POS2404 | His Trp(+) | 4.4798 | 2.2746 | 8.8230 | 0.0000 | 0.0000 |
| POS2678 | Stearoylcarnitine(+) | 2.7084 | 1.5519 | 4.7270 | 0.0005 | 0.0006 |
| POS2814 | LysoPE(0:0/18:2(9Z,12Z))(+) | 8.7970 | 3.7356 | 20.7160 | 0.0000 | 0.0000 |
| POS2819 | LysoPC(P-16:0)(+) | 0.0001 | 0.0000 | 0.0223 | 0.0009 | 0.0012 |
| POS2828 | LysoPE(0:0/18:0)(+) | 0.0094 | 0.0013 | 0.0681 | 0.0000 | 0.0000 |
| POS2829 | PC(O-16:0/0:0)(+) | 0.0001 | 0.0000 | 0.0289 | 0.0016 | 0.0020 |
| POS2870 | PC(P-17:0/0:0)(+) | 0.0001 | 0.0000 | 0.0172 | 0.0004 | 0.0006 |
| POS2876 | LysoPC(16:0)(+) | 0.0557 | 0.0169 | 0.1832 | 0.0000 | 0.0000 |
| POS2877 | PC(O-16:0/O-1:0)(+) | 0.0000 | 0.0000 | 0.0041 | 0.0004 | 0.0006 |
| POS2891 | LysoPE(0:0/20:5(5Z,8Z,11Z,14Z,17Z))(+) | 7.4490 | 3.3494 | 16.5666 | 0.0000 | 0.0000 |
| POS2897 | LysoPE(0:0/20:4(5Z,8Z,11Z,14Z))(+) | 4.3171 | 2.2365 | 8.3331 | 0.0000 | 0.0000 |
| POS2922 | LysoPC(P-18:0)(+) | 0.0001 | 0.0000 | 0.0181 | 0.0008 | 0.0011 |
| POS2930 | LysoPC(17:0)(+) | 0.0071 | 0.0009 | 0.0555 | 0.0000 | 0.0000 |
| POS2932 | LysoPC(O-18:0)(+) | 0.0000 | 0.0000 | 0.0175 | 0.0026 | 0.0033 |
| POS2936 | LysoPC(32:4(17Z,20Z,23Z,26Z))(+) | 0.0000 | 0.0000 | 0.0271 | 0.0028 | 0.0035 |
| POS2982 | LysoPC(18:1(9Z))(+) | 0.0855 | 0.0293 | 0.2488 | 0.0000 | 0.0000 |
| POS2999 | PC(O-16:0/2:0)(+) | 0.1324 | 0.0481 | 0.3649 | 0.0001 | 0.0002 |
| POS3001 | PC(17:1(10Z)/0:0)(+) | 0.1121 | 0.0425 | 0.2955 | 0.0000 | 0.0000 |
| POS3003 | Isodesmosine(+) | 0.0314 | 0.0074 | 0.1329 | 0.0000 | 0.0000 |
| POS3004 | LysoPE(0:0/22:6(4Z,7Z,10Z,13Z,16Z,19Z))(+) | 12.5807 | 4.2902 | 36.8925 | 0.0000 | 0.0000 |
| POS3013 | LysoPE(0:0/22:5(4Z,7Z,10Z,13Z,16Z))(+) | 2.7514 | 1.5502 | 4.8832 | 0.0005 | 0.0007 |
| POS3054 | PC(16:1(9Z)/2:0)(+) | 0.0307 | 0.0069 | 0.1364 | 0.0000 | 0.0000 |
| POS3056 | PC(O-16:0/3:1(2E))(+) | 0.0077 | 0.0011 | 0.0561 | 0.0000 | 0.0000 |
| POS3059 | PC(O-18:0/O-2:1(1E))(+) | 0.0001 | 0.0000 | 0.0424 | 0.0033 | 0.0040 |
| POS3109 | LysoPC(20:2(11Z,14Z))(+) | 0.0204 | 0.0043 | 0.0968 | 0.0000 | 0.0000 |
| POS3113 | LysoPC(20:1(11Z))(+) | 0.0038 | 0.0002 | 0.0628 | 0.0001 | 0.0002 |
| POS3148 | PC(16:0/2:0)(+) | 0.0097 | 0.0013 | 0.0753 | 0.0000 | 0.0000 |
| POS3198 | LysoPC(22:4(7Z,10Z,13Z,16Z))(+) | 0.0778 | 0.0273 | 0.2214 | 0.0000 | 0.0000 |
| POS326 | Indole(+) | 0.0006 | 0.0000 | 0.0119 | 0.0000 | 0.0000 |
| POS347 | L-Homoserine(+) | 0.0012 | 0.0001 | 0.0218 | 0.0000 | 0.0000 |
| POS453 | Pyroglutamic acid(+) | 0.0020 | 0.0001 | 0.0326 | 0.0000 | 0.0000 |
| POS512 | L-Aspartate(+) | 0.0000 | 0.0000 | 0.0019 | 0.0000 | 0.0000 |
| POS660 | L-Glutamine(+) | 3.4892 | 1.9488 | 6.2473 | 0.0000 | 0.0001 |
| POS661 | L-Lysine(+) | 0.0000 | 0.0000 | 0.0006 | 0.0000 | 0.0000 |
| POS668 | L-Glutamate(+) | 0.0039 | 0.0004 | 0.0354 | 0.0000 | 0.0000 |
| POS839 | L-Carnitine(+) | 10.1246 | 4.0933 | 25.0427 | 0.0000 | 0.0000 |
| POS884 | L-Methionine S-oxide (+) | 0.0000 | 0.0000 | 0.0114 | 0.0004 | 0.0006 |
| POS886 | L-Phenylalanine(+) | 0.0012 | 0.0001 | 0.0170 | 0.0000 | 0.0000 |
| *Potential biomarkers were adjusted with significant clinical index (Age, BMI, WC,Waist–hip ratiourine albumin,HDL-C,LDL-C,TC, GGT,Glu0,Glu120,HbA1C) discriminating Pre-diabetes group from T2D group by multilogistic regression analysis. | | | | | | |
|  |  |  |  |  |  |  |

**Supplementary Table S6. Random forest classification based on untargeted metabolomics profiling.**

| **Clinical index/metabolites*** | **0** | **1** | **MeanDecreaseAccuracy** | **MeanDecreaseGini** |
| --- | --- | --- | --- | --- |
| Waist-hip.ratio | 0.0340 | 0.0672 | 0.0502 | 19.1242 |
| WC | 0.0032 | 0.0317 | 0.0170 | 11.2854 |
| BMI | 0.0025 | 0.0309 | 0.0165 | 12.2127 |
| TC | 0.0031 | 0.0150 | 0.0089 | 11.0027 |
| GGT | 0.0056 | 0.0007 | 0.0031 | 10.6154 |
| LDL-C | -0.0043 | 0.0054 | 0.0004 | 9.0218 |
| HDL-C | -0.0072 | 0.0063 | -0.0007 | 9.1378 |
| Urine-albumin | -0.0059 | -0.0027 | -0.0044 | 8.0904 |
| PC(O-16:0/O-1:0)(+) | 0.0045 | 0.0400 | 0.0216 | 5.0265 |
| PC(O-18:0/O-2:1(1E))(+) | 0.0162 | 0.0215 | 0.0186 | 3.8810 |
| Phe Phe(+) | 0.0098 | 0.0260 | 0.0177 | 4.9094 |
| Carvacrol (-) | 0.0159 | 0.0198 | 0.0176 | 2.5261 |
| Inosine (-) | 0.0108 | 0.0229 | 0.0168 | 2.4967 |
| 1-Palmitoyl Lysophosphatidic Acid(-) | 0.0138 | 0.0184 | 0.0156 | 3.3382 |
| PE(O-18:1(9Z)/0:0)(-) | 0.0028 | 0.0289 | 0.0152 | 3.3713 |
| PC(P-17:0/0:0)(+) | 0.0061 | 0.0226 | 0.0145 | 3.5042 |
| LysoPC(P-16:0)(+) | 0.0095 | 0.0183 | 0.0140 | 3.5747 |
| PC(O-16:0/3:1(2E))(+) | 0.0107 | 0.0145 | 0.0123 | 2.2652 |
| L-Histidine(-) | 0.0063 | 0.0181 | 0.0117 | 1.9871 |
| PE(P-16:0e/0:0)(-) | 0.0062 | 0.0166 | 0.0110 | 3.5543 |
| LysoPC(20:1(11Z))(+) | 0.0079 | 0.0147 | 0.0108 | 2.8784 |
| LysoPC(P-18:0)(+) | 0.0044 | 0.0155 | 0.0096 | 3.0456 |
| His Trp(+) | 0.0034 | 0.0157 | 0.0093 | 2.1422 |
| LysoPC(20:2(11Z,14Z))(+) | 0.0061 | 0.0090 | 0.0077 | 2.2895 |
| L-Methionine S-oxide (+) | 0.0035 | 0.0124 | 0.0076 | 1.4493 |
| PG(P-20:0/0:0)(-) | 0.0057 | 0.0091 | 0.0074 | 2.2648 |
| Prostaglandin B1(-) | 0.0023 | 0.0127 | 0.0071 | 0.8688 |
| LysoPC(O-18:0)(+) | 0.0023 | 0.0109 | 0.0066 | 1.9150 |
| L-Carnitine(+) | 0.0063 | 0.0070 | 0.0066 | 1.0730 |
| PI(O-18:0/18:4(6Z,9Z,12Z,15Z))(-) | 0.0021 | 0.0105 | 0.0063 | 0.7761 |
| PC(O-16:0/0:0)(+) | 0.0028 | 0.0088 | 0.0058 | 2.6399 |
| PI(O-16:0/20:5(5Z,8Z,11Z,14Z,17Z))(-) | 0.0044 | 0.0073 | 0.0058 | 0.4788 |
| PC(16:1(9Z)/2:0)(+) | 0.0000 | 0.0110 | 0.0056 | 0.3929 |
| LysoPC(32:4(17Z,20Z,23Z,26Z))(+) | 0.0042 | 0.0069 | 0.0056 | 1.2191 |
| LysoPE(0:0/18:2(9Z,12Z))(+) | 0.0036 | 0.0066 | 0.0050 | 0.9616 |
| LysoPE(0:0/20:5(5Z,8Z,11Z,14Z,17Z))(+) | 0.0055 | 0.0049 | 0.0050 | 1.0378 |
| PG(22:1(11Z)/0:0)(-) | 0.0055 | 0.0045 | 0.0049 | 0.6338 |
| LysoPE(0:0/18:2(9Z,12Z))(-) | 0.0060 | 0.0037 | 0.0048 | 0.9249 |
| Eicosapentaenoic acid(-) | 0.0037 | 0.0058 | 0.0047 | 0.9515 |
| PE(O-16:0/0:0)(-) | 0.0028 | 0.0053 | 0.0041 | 1.1733 |
| LysoPE(0:0/22:6(4Z,7Z,10Z,13Z,16Z,19Z))(+) | 0.0020 | 0.0060 | 0.0040 | 0.3674 |
| Pyroglutamic acid(+) | 0.0004 | 0.0074 | 0.0038 | 0.3666 |
| LysoPC(16:0)(+) | 0.0013 | 0.0063 | 0.0037 | 0.4247 |
| LysoPC(17:0)(+) | -0.0001 | 0.0071 | 0.0035 | 0.4986 |
| PC(16:0/2:0)(+) | 0.0045 | 0.0022 | 0.0034 | 0.5244 |
| PE(O-18:0/0:0)(-) | 0.0018 | 0.0049 | 0.0033 | 0.4579 |
| Stearoylcarnitine(+) | -0.0008 | 0.0077 | 0.0033 | 0.4335 |
| PI(16:0/20:4(5Z,8Z,11Z,14Z))(-) | 0.0004 | 0.0061 | 0.0032 | 0.3023 |
| L-Aspartate(+) | 0.0020 | 0.0043 | 0.0031 | 0.6997 |
| LysoPE(0:0/18:1(11Z))(-) | 0.0028 | 0.0031 | 0.0030 | 0.6531 |
| L-Serine(+) | 0.0019 | 0.0037 | 0.0029 | 0.7958 |
| L-Glutamine(+) | 0.0037 | 0.0018 | 0.0028 | 0.2577 |
| Glu Gln(+) | 0.0004 | 0.0051 | 0.0028 | 0.3813 |
| Gamma-Glu-Leu(-) | 0.0032 | 0.0022 | 0.0027 | 0.2777 |
| Citrate(-) | -0.0010 | 0.0065 | 0.0026 | 0.4614 |
| 13(S)-HODE(-) | 0.0016 | 0.0035 | 0.0024 | 0.6359 |
| L-Phenylalanine(+) | 0.0004 | 0.0045 | 0.0024 | 0.5384 |
| Decanoylcarnitine(+) | 0.0016 | 0.0033 | 0.0024 | 0.3417 |
| 3-Indolepropionic acid(+) | 0.0030 | 0.0017 | 0.0023 | 0.1985 |
| L-Phenylalanine(-) | 0.0009 | 0.0035 | 0.0023 | 0.3186 |
| LysoPE(0:0/22:5(4Z,7Z,10Z,13Z,16Z))(-) | -0.0001 | 0.0045 | 0.0023 | 0.1975 |
| PC(O-16:0/2:0)(+) | 0.0029 | 0.0019 | 0.0023 | 0.3364 |
| PG(O-18:0/0:0)(-) | 0.0020 | 0.0023 | 0.0022 | 0.7754 |
| Isodesmosine(+) | -0.0003 | 0.0047 | 0.0022 | 0.2798 |
| PI(O-16:0/18:3(9Z,12Z,15Z))(-) | 0.0012 | 0.0027 | 0.0019 | 0.2193 |
| L-Glutamate(+) | 0.0020 | 0.0018 | 0.0019 | 0.2096 |
| LysoPE(0:0/22:6(4Z,7Z,10Z,13Z,16Z,19Z))(-) | 0.0019 | 0.0021 | 0.0019 | 0.4612 |
| PS(O-20:0/0:0)(-) | 0.0011 | 0.0027 | 0.0019 | 0.4995 |
| L-Aspartate(-) | 0.0004 | 0.0035 | 0.0019 | 0.1908 |
| Leu His(+) | 0.0000 | 0.0037 | 0.0019 | 0.2689 |
| Guanosine (-) | 0.0013 | 0.0025 | 0.0018 | 0.4614 |
| Glu Arg(+) | 0.0000 | 0.0036 | 0.0017 | 0.1724 |
| LysoPC(18:1(9Z))(+) | -0.0003 | 0.0037 | 0.0017 | 0.1674 |
| Glu Glu(+) | 0.0014 | 0.0019 | 0.0017 | 0.1928 |
| LysoPE(0:0/20:3(11Z,14Z,17Z))(-) | 0.0006 | 0.0028 | 0.0016 | 0.1723 |
| 5-HEPE(-) | 0.0012 | 0.0020 | 0.0016 | 0.4845 |
| LysoPC(22:4(7Z,10Z,13Z,16Z))(+) | 0.0013 | 0.0013 | 0.0013 | 0.1361 |
| Gamma-Glu-Leu(+) | 0.0009 | 0.0017 | 0.0013 | 0.3036 |
| Indole(+) | 0.0015 | 0.0009 | 0.0012 | 0.1791 |
| Cholesterol sulfate(-) | 0.0010 | 0.0015 | 0.0012 | 0.3898 |
| PG(P-18:0/0:0)(-) | 0.0003 | 0.0022 | 0.0012 | 0.2726 |
| LysoPE(0:0/20:4(5Z,8Z,11Z,14Z))(+) | 0.0008 | 0.0015 | 0.0011 | 0.1609 |
| LysoPE(0:0/22:5(4Z,7Z,10Z,13Z,16Z))(+) | 0.0008 | 0.0015 | 0.0011 | 0.1969 |
| LysoPE(18:0/0:0)(-) | 0.0011 | 0.0012 | 0.0011 | 0.2240 |
| L-Lysine(+) | -0.0001 | 0.0023 | 0.0011 | 0.1068 |
| PI(18:0/0:0)(-) | -0.0001 | 0.0023 | 0.0010 | 0.2682 |
| PG(22:4(7Z,10Z,13Z,16Z)/0:0)(-) | 0.0000 | 0.0020 | 0.0010 | 0.3706 |
| 7-HDoHE(-) | 0.0012 | 0.0004 | 0.0009 | 0.2803 |
| PI(16:0/0:0)(-) | 0.0010 | 0.0009 | 0.0009 | 0.2311 |
| Sphingosine(+) | 0.0000 | 0.0016 | 0.0008 | 0.2984 |
| Phe Glu(+) | 0.0005 | 0.0012 | 0.0008 | 0.2534 |
| 2-hydroxyhexadecanoic acid(-) | 0.0005 | 0.0011 | 0.0008 | 0.1615 |
| LysoPE(0:0/18:0)(+) | -0.0005 | 0.0022 | 0.0008 | 0.1609 |
| L-Glutamate(-) | 0.0001 | 0.0011 | 0.0006 | 0.1773 |
| Glu Lys(+) | 0.0008 | 0.0004 | 0.0005 | 0.2329 |
| L-Glutamine(-) | 0.0007 | 0.0005 | 0.0005 | 0.1905 |
| PI(18:1(9Z)/0:0)(-) | 0.0005 | 0.0004 | 0.0004 | 0.1418 |
| 5-Methoxytryptophol(-) | 0.0002 | 0.0006 | 0.0003 | 0.0983 |
| PI(14:0/22:1(11Z))(-) | 0.0004 | 0.0002 | 0.0003 | 0.3081 |
| L-Tyrosine(-) | -0.0002 | 0.0008 | 0.0003 | 0.0828 |
| PC(17:1(10Z)/0:0)(+) | -0.0001 | 0.0006 | 0.0003 | 0.1745 |
| Ser Leu(+) | -0.0001 | 0.0007 | 0.0002 | 0.1658 |
| PS(20:2(11Z,14Z)/0:0)(-) | 0.0003 | 0.0002 | 0.0002 | 0.1209 |
| PG(20:2(11Z,14Z)/0:0)(-) | -0.0002 | 0.0002 | 0.0000 | 0.2421 |
| L-Tyrosine(+) | -0.0007 | 0.0002 | -0.0002 | 0.1452 |
| Gamma-Glutamyltyrosine(+) | -0.0001 | -0.0003 | -0.0002 | 0.2296 |
| L-Homoserine(+) | 0.0000 | -0.0004 | -0.0002 | 0.1727 |
| PI(16:0/18:2(9Z,12Z))(-) | -0.0006 | -0.0004 | -0.0005 | 0.1787 |
| PI(16:0/18:1(11Z))(-) | -0.0001 | -0.0013 | -0.0007 | 0.2506 |
| *Clinical index and metabolites selected by randomforest model discriminating Pre-diabetes group from T2D group | | | | |
