# Supplementary material for: Potential Novel Serum Metabolic Markers Associated With Progression of Prediabetes to Overt Diabetes in a Chinese Population
Source: Front Endocrinol (Lausanne). 2022 Jan 5;12:745214. doi: 10.3389/fendo.2021.745214 (PMC8766640; doi:10.3389/fendo.2021.745214)
Supplement: Supplementary file 3 [file DataSheet_3.docx]

***Metabolites preparation and extraction***

The collected serum samples were thawed on ice, and metabolites were extracted with methanol using a previously described method (Dunn, W. B, et al. Procedures for large-scale metabolic profiling of serum and plasma using gas chromatography and liquid chromatography coupled to mass spectrometry. Nature Protoc.2011, 6(7), 1060−1083.). The low-molecular-weight metabolites (<1000 Da) in the serum samples were isolated and treated as follows. First, 50 μL of thawed serum samples were collected and then precipitated by 150 μL of methanol, and 10 μL of 1 mg/mL 2-Chloro-L-phenylalanine as internal standard. After centrifugation at 14000 g for 10 min at 4 °C, the supernatant was transferred to a 1.5 mL sample vail and 1 μL of the supernatant was injected into the UHPLC−MS. A “quality control” (QC) sample was also prepared by mixing equal volumes (10 μL) from each serum sample before sample preparation as they were aliquoted for analysis. This amalgamative sample was used to estimate a “mean” profile representing all the analytes encountered during the analysis. The samples were randomly ordered, and 10 QC samples were initially injected to condition the column. One QC sample was injected and analyzed every 10 samples to investigate the repeatability of the data (Want EJ, et al. Global metabolic profiling procedures for urine using UPLC-MS. Nat Protoc 2010;5: 1005–18.).

***Ultra high performance liquid chromatography–mass spectrometry method for metabolomics***

Serum samples were analyzed through using an UHPLC system (1290, Agilent Technologies) with a UPLC HSS T3 column (2.1 mm × 100 mm, 1.8 μm, Waters) coupled to Q Exactive Focus (Thermo Fisher Scientific, MA, USA) via a previously described method (Jialin Wang, et al. Serum metabolomics for early diagnosis of esophageal squamous cell carcinoma by UHPLC-QTOF/MS. Metabolomics (2016)12:116.) with modifications. The mobile phase A was 0.1% formic acid in water for positive, and 0.5 mmol/L ammonium fluoride in water for negative, and the mobile phase B was acetonitrile. The elution gradient was set as follows: 0 min, 1% B; 1 min, 1% B; 8 min, 99% B; 10 min, 99% B; 10.1 min, 1% B; 12 min, 1% B. The flow rate was 0.5 mL/min. The column temperature was set at 35 °C. The auto-sampler temperature was set at 4 °C and the injection volume was 1 μL. The QE mass spectrometer was used for its ability to acquire MS/MS spectra on an information-dependent basis (IDA) during an LC/MS experiment. In this mode, the acquisition software (Xcalibur v4.0.27, Thermo) continuously evaluates the full scan survey MS data as it collects and triggers the acquisition of MS/MS spectra depending on preselected criteria. ESI source conditions were set as following: Sheath gas flow rate as 45 Arb, Aux gas flow rate as 15 Arb, Capillary temperature 320 °C and Spray Voltage as 3.5 kV (positive) or -3.1 kV (negative), respectively. For MS1 full scan, the parameters were set as following: resolution as 70000, AGC target as 1e6. And for MS/MS, resolution as 17500, isolation window as 2.0 m/z, AGC target as 5e4, Collision energy as 20/40/60 eV in NCE model.

***Acquisition of the high-quality non-targeted metabolic profile and metabolite identification***

The acquired MS data pretreatments included peak selection and grouping, retention time correction, second peak grouping, and isotopes and adducts annotation, were performed as previously described with a few modifications. LC-MS raw data files were converted into *mzXML* format using the “msconvert” program from ProteoWizard (v3.0.6526) and then analyzed by the XCMS (Tautenhahn et al. Highly sensitive feature detection for high resolution LC/MS. BMC Bioinformatics. 2008 Nov 28;9:504) and CAMERA toolbox (Kuhl et al. CAMERA: An integrated strategy for compound spectra extraction and annotation of liquid chromatography/mass spectrometry data sets. Anal Chem. 2012 Jan 3;84(1):283-9.) with R statistical language (*v*3.5.3). The CentWave algorithm in XCMS was used for peak detection. The parameter “peak-width” was set as (5, 20) in units of seconds, referring to the minimum and maximum peak widths for peak detection. The parameter ‘‘snthresh’’ is set as 3 for sensitive peak detection. For multiple LC–MS data files, an ordered bijective interpolated warping (OBI-Warp) algorithm in XCMS was used for peak alignment (Prince JT, Marcotte EM. Chromatographic alignment of ESI-LC-MS proteomics data sets by ordered bijective interpolated warping. Anal Chem. 2006 Sep 1;78(17):6140-52.). The CAMERA package is used for peak annotation. By using retention time and the m/z data pairs as the identifiers for each ion, we obtained ion intensities of each peak and generated three dimensional matrix containing arbitrarily assigned peak indices (retention time-*m*/*z* pairs), ion intensities (variables) and sample names (observations).

The matrix was further reduced by removing peaks with missing values (ion intensity = 0) in more than 50% samples and 20% QC samples and those with isotope ions from each group to obtain consistent variables. Each retained peak was then normalized to the QC sample using Robust Loess Signal Correction (R-LSC) ( Dunn, W. B, et al. Procedures for large-scale metabolic profiling of serum and plasma using gas chromatography and liquid chromatography coupled to mass spectrometry. Nature Protoc.2011, 6(7), 1060−1083.) on the basis of the periodic analysis of the QC sample and the true samples to ensure the data of high quality within an analytical run, which is accepted as a quality assurance strategy in metabolic profiling. The relative standard deviation (RSD) value of metabolites in the QC samples was set at a threshold of 30%, as a standard in the assessment of repeatability in metabolomics data sets.

***Metabolites annotations and identifications***

Exact molecular mass data (m/z) from significant peaks were used to search the online HMDB database (<http://www.hmdb.ca>) (Wishart, D. S. et al. HMDB 3.0—The Human Metabolome Database in 2013. Nucleic Acids Res 41, D801–D807 (2013).) and KEGG database ([www.genome.jp/kegg/](http://www.genome.jp/kegg/)) (Kanehisa, M. et al. KEGG: Kyoto Encyclopedia of Genes and Genomes. Nucleic Acids Res 28, 27–30 (2000).) for metabolite identification. A metabolite name was reported when a mass difference between observed and theoretical mass was <10 ppm. Isotopic distribution measurements were used to further validate the metabolite molecular formula of matched metabolites. The identities of significant metabolites were confirmed by comparison of their MS/MS spectra and retention time with those commercially available reference standards.

***PERMANOVA for the influence of clinical and lifestyle factors***

Permutation multivariate analysis of variance (PERMANOVA), a permutation-based version of the multivariate analysis of variance, was employed to test the statistical significant differences between metabolic profiles and individuals’ phenotypes (Anderson, M. J. A new method for non-parametric multivariate analysis of variance. Aust. Ecol.2001, 26(1), 32−46.). PERMANOVA analysis was performed on the normalized metabolite profiles and phenotypes with Bray–Curtis distance (adonis function, “vegan” package in R). The number of permutations was 999. The P-value was corrected for multiple tests using an FDR (Benjamini–Hochberg) cut-off of 0.05.
