# Supplementary figures and images for: Potential Novel Serum Metabolic Markers Associated With Progression of Prediabetes to Overt Diabetes in a Chinese Population
Source: Front Endocrinol (Lausanne). 2022 Jan 5;12:745214. doi: 10.3389/fendo.2021.745214 (PMC8766640; doi:10.3389/fendo.2021.745214)

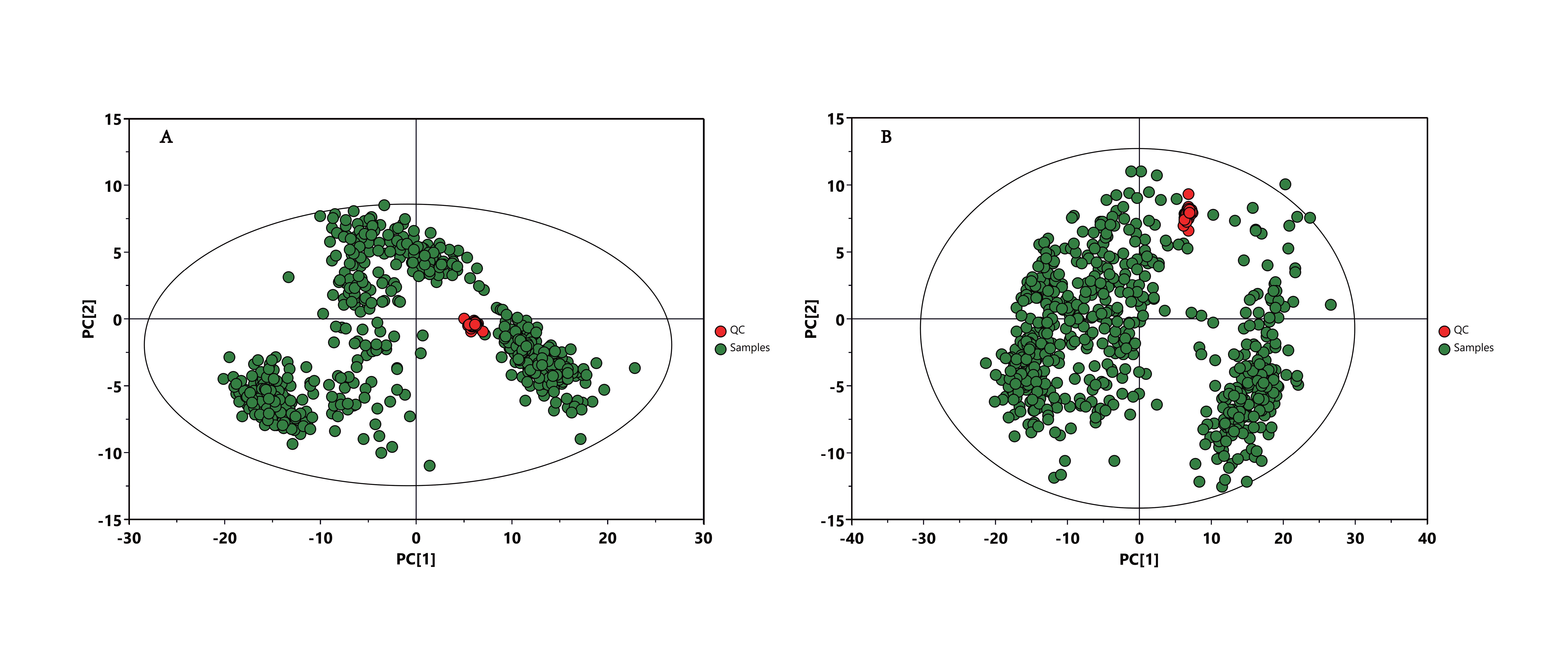

Supplement: Supplementary file 4 [file Image_1.tiff]

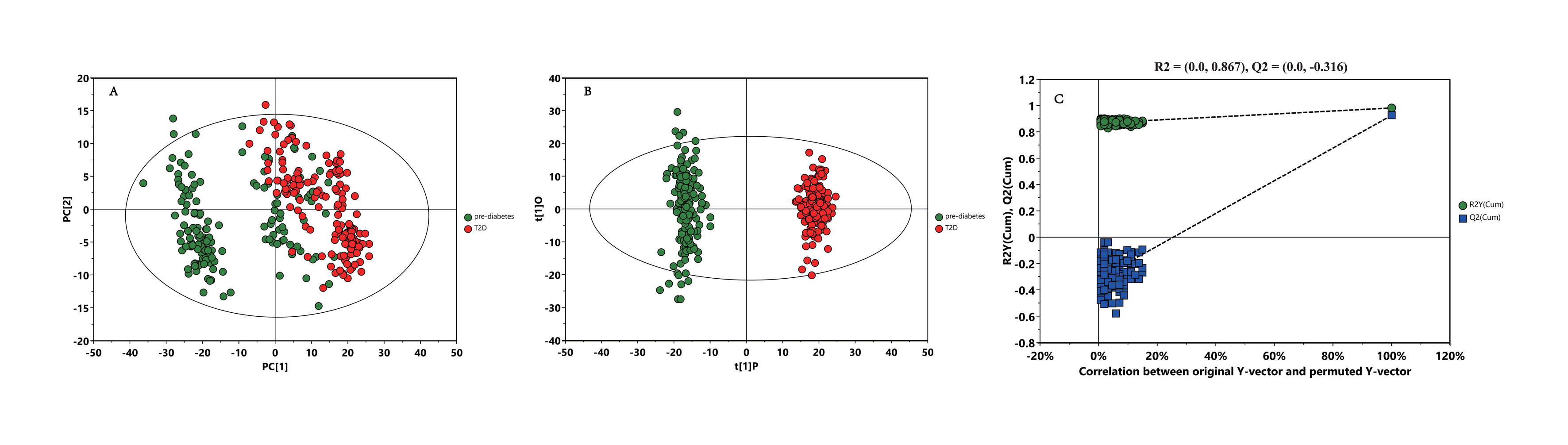

Supplement: Supplementary file 5 [file Image_2.tif]
